# Diabetes Mellitus Facilitates Gallstone Formation Through CXCR2‐NETs–Mediated Liver‐Bile Barrier Damage

**DOI:** 10.1002/advs.202519500

**Published:** 2026-02-16

**Authors:** Chao Shi, Shuo Feng, Tianming Liu, Ziang Meng, Yi Zheng, Zhenghao Huang, Tong Wang, Bojian Zhang, Dongbo Xue, Biao Ma, Xianzhi Meng

**Affiliations:** ^1^ Department of General Surgery The First Affiliated Hospital of Harbin Medical University Harbin China; ^2^ Key Laboratory of Hepatosplenic Surgery Ministry of Education, The First Affiliated Hospital of Harbin Medical University Harbin China

**Keywords:** CXCR2, diabetes, gallstone, NETs

## Abstract

**Background**: Gallstone disease is a major health burden worldwide, and diabetes is believed to increase the risk of gallstone formation. This study investigates how diabetes promotes gallstone formation and elucidates the underlying mechanism.

**Methods**: Data from the National Health and Nutrition Examination Survey and Mendelian randomization analyses were used to identify the causal relationship between diabetes and gallstones. Through the integration of multi‐omics sequencing and molecular dynamics simulation, CXCR2 was identified as a molecular bridge linking both diseases. Animal models were established, and Western blotting, reverse transcription quantitative polymerase chain reaction, and enzyme‐linked immunosorbent assay were employed to explore the relevant mechanisms.

**Results**: Clinical data demonstrated that diabetes is an independent risk factor for gallstones. Animal experiments revealed that diabetes upregulated the expression of CXCR2 in hepatic neutrophils, thereby promoting the formation of neutrophil extracellular traps (NETs). Excessive NETs disrupted the tight junctions between hepatocytes, allowing NETs to enter the bile and accelerate gallstone formation. Moreover, sarcosine inhibited CXCR2 expression, reduced NETs production, and decreased gallstone formation.

**Conclusions**: Diabetes promotes NETs formation through CXCR2 activation, which damages the liver‐bile barrier and facilitates gallstone development. Sarcosine may serve as a potential therapeutic agent, providing a theoretical basis for clinical intervention.

## Graphical abstract

Diabetes is an independent risk factor for gallstones. It upregulates CXCR2 expression in hepatic neutrophils, stimulating the formation of NETs that disrupt hepatocellular tight junctions and the liver‐bile barrier. NETs enter bile to accelerate gallstone development, while sarcosine inhibits CXCR2 and NETs production, effectively reducing diabetes‐induced gallstones and holding potential for future clinical application.

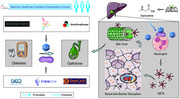

## 1 Background

Gallstone disease constitutes a major health concern in both developed and developing countries, affecting approximately 10%–15% of the adult population and accounting for a substantial number of hospital admissions [1]. It is widely acknowledged to be associated with multiple risk factors, including dietary habits, lifestyle, genetic predisposition, age, and gender [2]. Notably, its complications can lead to systemic symptoms and signs. Although cholecystectomy remains an effective treatment for gallstones, data from the United States in the early 2000s indicated that the overall mortality rate associated with gallstone disease continued to show an upward trend, and the disease imposed considerable economic burdens [3].

Diabetes mellitus is a prevalent metabolic disorder characterized by chronic hyperglycemia. Its primary etiology involves insufficient insulin secretion or impaired insulin action, which can damage multiple organs and systems throughout the body and lead to severe complications [4]. In 2021, an estimated 537 million adults worldwide were living with diabetes, and projections suggest that by 2050, this number will reach one billion [5]. The pathogenesis of diabetes is complex and typically arises from the combined effects of genetic and environmental factors, with significant etiological variations among different types of diabetes [6]. Therefore, diabetes mellitus is regarded as a common metabolic disorder that poses substantial challenges in clinical management.

Interestingly, gallstone disease is frequently regarded as being closely associated with metabolic syndrome, obesity, and diabetes mellitus. Notably, certain factors, such as a high‐fat diet, can contribute to the development of both diabetes and gallstones [7]. A clinical observational study conducted by Luthra et al. reported that diabetic patients experienced more intraoperative adverse events during laparoscopic cholecystectomy than non‐diabetic patients [8]. These findings prompted us to hypothesize that shared underlying mechanisms may exist between these two diseases and that targeting specific genes or metabolites might offer a potential therapeutic strategy for both conditions. Comorbidity analysis refers to the investigation of individuals who simultaneously suffer from two or more chronic diseases. In recent years, this field has gained growing attention in medical research, particularly among the elderly population, and plays an important role in the prevention and control of chronic diseases [9]. By integrating population data with experimental analyses, our study demonstrated that diabetes mellitus promotes the formation of neutrophil extracellular traps (NETs) through CXCR2 induction. This process subsequently disrupts the liver–bile barrier and facilitates gallstone formation, thereby providing a novel perspective for the treatment of these two metabolic disorders.

## 2 Methods

### 2.1 Study Design and Participants in NHANES

**FIGURE 1:**
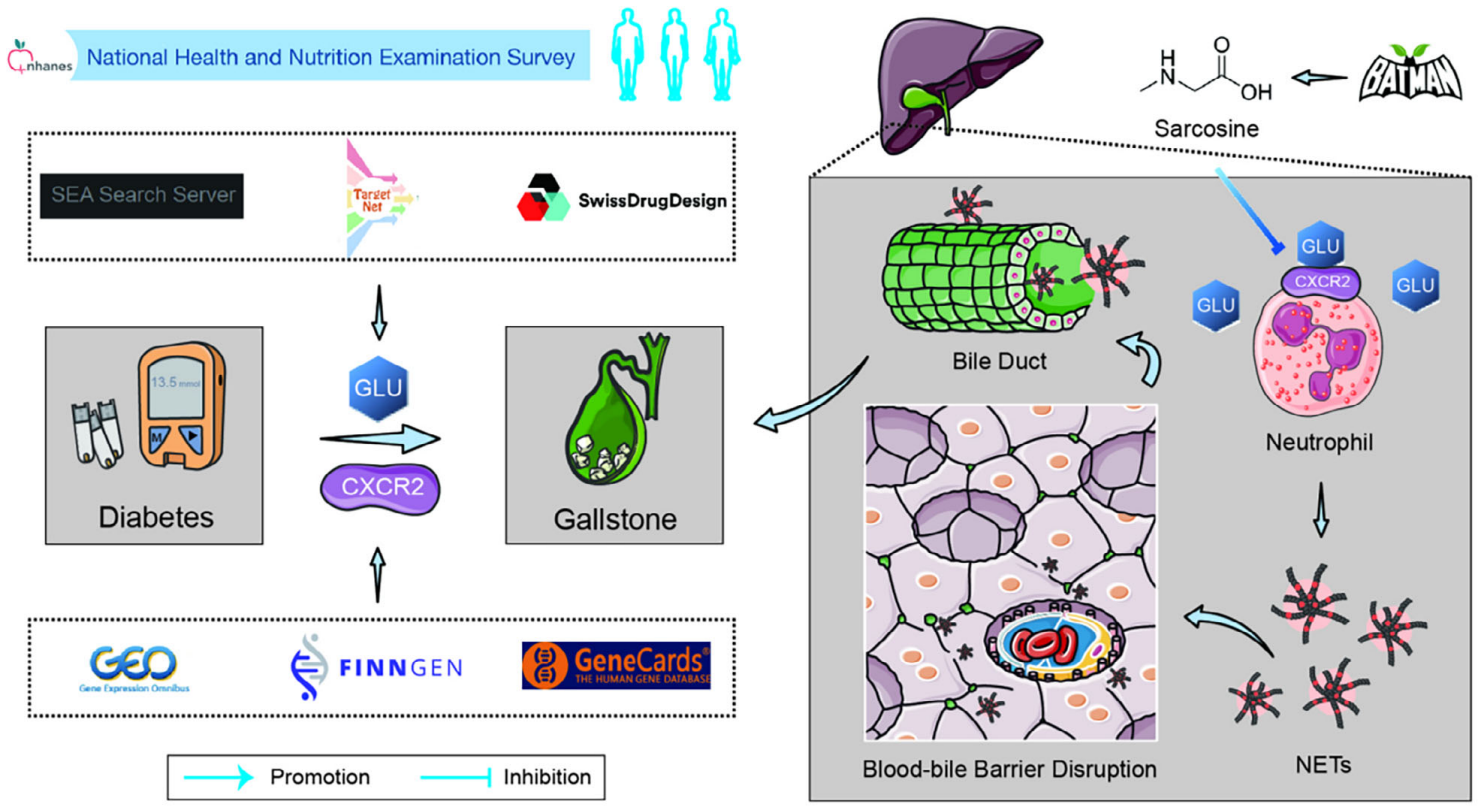
Overall concept map. Integrated multi‐database analysis revealed that diabetes is a risk factor for gallstone formation and that a causal relationship exists between them. Mouse experiments further confirmed that diabetes promotes the onset of gallstones. CXCR2 was identified as a key gene through which diabetes facilitates gallstone development. Glucose binds to CXCR2, leading to the upregulation of CXCR2 protein expression. The elevated expression of CXCR2 in neutrophils promotes the formation of NETs in the liver. Simultaneously, diabetes damages the liver–bile barrier by enhancing NETs formation in the liver, thereby enabling the release of NETs into the bile and exacerbating gallstone formation.

The National Health and Nutrition Examination Survey (NHANES) is a large‐scale, cross‐sectional study designed to assess the health and nutritional status of children and adults in the United States. Initiated in 1960, the survey primarily collects data through structured interviews and medical examinations (for details, see https://www.cdc.gov/nchs/nhanes/). For the present study, 10 652 adult participants were initially selected from the 2017–2020 NHANES database. A total of 456 participants were excluded due to missing gallstone detection data, and 6224 were excluded due to incomplete information on other covariates. Consequently, 3972 participants were included in the final analysis (Figure 1). All data were weighted in accordance with the NHANES analytical guidelines, ensuring that the study sample was representative of a U.S. population of 77 484 080 individuals.

### 2.2 Definition of Variables in NHANES

Sociodemographic and lifestyle data were collected using a professionally standardized household questionnaire, which included variables such as age, sex, ethnicity, gallstone status, educational level, marital status, smoking behavior, and alcohol consumption. All laboratory tests were conducted by trained medical personnel at mobile examination centers. Gallstone disease was identified through a standardized clinical interview question: “Have you ever been told by a doctor or other health professional that you have gallstones?” Body mass index (BMI) was calculated as weight in kilograms divided by height in meters squared (kg/m^2^). The diagnosis of diabetes mellitus was determined based on participants’ medical history and blood glucose measurements [10]. The metabolic score for insulin resistance (METS‐IR), homeostatic model assessment for insulin resistance (HOMA‐IR), and stress hyperglycemia ratio (SHR) were all computed in accordance with methods described in previous studies [11, 12, 13].

### 2.3 Mendelian Randomization (MR) Analysis

Five statistical methods were employed in this study: inverse variance weighted (IVW), weighted median, MR‐Egger, simple mode, and weighted mode. Among these, the IVW method—the most widely used approach in MR analyses—served as the primary analytical technique in the present study [14]. However, it is important to note that horizontal pleiotropy of single‐nucleotide polymorphisms (SNPs) in the IVW model may compromise the reliability of the results. To address this limitation and enhance the robustness of the primary analysis, the four additional methods mentioned above were jointly applied. The weighted median method maintained statistical power comparable to that of the IVW approach while tolerating up to 50% of invalid SNPs. Although the MR‐Egger method had relatively lower statistical power, it could provide unbiased causal estimates when pleiotropy occurred in MR analyses [15]. The mode‐based methods (simple and weighted mode) were used to assess the causal effects of individual SNPs and perform clustering: the simple mode method identified the largest SNP cluster to determine causal estimates, whereas the weighted mode method assigned weights to SNPs to further enhance the robustness of causal inference. Finally, the results of the MR analysis were visualized using a forest plot.

### 2.4 Mendelian Randomization Study Based on Druggable Genes

Following methodologies established in previous studies [16], we obtained *cis*‐expression quantitative trait loci (*cis‐*eQTLs) of druggable genes from the eQTLGen Consortium, which were used as the exposure variable. For the outcome variable, genome‐wide association study data for gallstones were retrieved from the Finnish database (GCST90436382). MR analysis was then conducted to simulate the potential effects of drugs targeting these druggable genes on gallstone development. Subsequently, colocalization analysis was performed to estimate the probability that the *cis*‐eQTLs of druggable genes and gallstone traits share the same causal genetic variants.

### 2.5 Network Toxicological Analysis of Glucose

The simplified molecular input line entry system sequence of the investigational compound (glucose) was first retrieved from the PubChem database [17] (https://pubchem.ncbi.nlm.nih.gov/). Subsequently, network toxicology‐based target prediction for glucose was conducted using three databases: TargetNet (http://targetnet.scbdd.com), SwissTargetPrediction (http://www.swisstargetprediction.ch/), and SEA (https://sea.bkslab.org/). Gallstone‐related target libraries were primarily constructed using the GeneCards database [18] (https://www.genecards.org/), with searches performed using the keywords “gallstone” and “cholelithiasis.” Finally, all identified targets from the databases were merged and deduplicated.

### 2.6 Molecular Dynamics Simulation (MDS)

MDS were performed using the GROMACS 2022 software package. The general Amber force field was applied to small molecules, while the AMBER14SB force field and the TIP3P water model were used for proteins. The protein and small‐molecule ligand files were merged to construct the simulation system of the complex. All simulations were conducted under isothermal–isobaric (NPT) and periodic boundary conditions. During the simulations, all bonds involving hydrogen atoms were constrained using the LINCS algorithm, with an integration time step of 2 fs. Electrostatic interactions were calculated using the particle mesh Ewald method with a cutoff distance of 1.2 nm. For non‐bonded interactions, the cutoff distance was set to 10 Å, and neighbor lists were updated every 10 steps.

The V‐rescale temperature‐coupling method was employed to maintain the system temperature at 298 K, while the Berendsen barostat was used to control the pressure at 1 bar. Prior to the production run, equilibration was performed for 100 ps under the NVT (canonical) ensemble and 100 ps under the NPT ensemble, both at 298 K. Subsequently, a 100 ns production simulation was carried out for the complex system, with conformations recorded every 10 ps. After the simulation, VMD and PyMOL software were used to analyze the simulation trajectories. Finally, the g_mmpbsa program was applied to calculate the perform MMPBSA (molecular mechanics Poisson–Boltzmann surface area) binding free energy between the protein and the small‐molecule ligand using the Molecular Mechanics Poisson–Boltzmann Surface Area approach.

### 2.7 Surface Plasmon Resonance (SPR) Detection

#### 2.7.1 Immobilization of CXCR2

The recombinant CXCR2 protein was immobilized onto a Series S CMS sensor chip (Cytiva) using a standard amine‐coupling procedure. Briefly, the sensor chip surface was activated for 240 s by injecting a freshly prepared mixture of 400 mm 1‐ethyl‐3‐(3‐dimethylaminopropyl) carbodiimide(EDC) and 100 mM N‐hydroxysuccinimide(NHS) at a flow rate of 20 µL/min. CXCR2 was diluted to 50 µg/mL in the immobilization buffer (10 mm sodium acetate, pH 5.0) and injected over the activated surface at a flow rate of 20 µL/min until the desired immobilization level(RU) was achieved. The remaining activated ester groups on the chip surface were then blocked by injecting 1 M ethanolamine hydrochloride (pH 8.5) for 240 s at the same flow rate. A reference flow cell was activated and blocked similarly but without protein injection, for background subtraction.

Twofold serial dilutions of glucose (Analyte 1) were prepared in the running buffer (HBS‐EP+, 10 mm HEPES, 150 mm NaCl, 3 mm EDTA, 0.05% v/v surfactant P20, pH 7.4) to obtain eight concentrations ranging from 0.39 to 100 µm. Each glucose solution was injected over the CXCR2‐immobilized and reference surfaces at a flow rate of 20 µL/min for an association phase of 240 s, followed by a 360‐s dissociation phase with running buffer. The samples were injected in ascending order of concentration. After each cycle, the sensor chip surface was regenerated by injecting 10 mm glycine‐HCl (pH 2.0) for 10 s at a flow rate of 150 µL/min to completely remove the bound glucose. The response from the reference flow cell was subtracted from the response in the CXCR2‐immobilized flow cell to yield the specific binding signal.

### 2.8 Clinical Sample Collection

All human and animal experiments were approved by the Ethics Committee of the First Affiliated Hospital of Harbin Medical University (Nos. 2025395 and 2024081), and written informed consent was obtained from all human participants. Liver tissue, gallbladder tissue, serum, and bile samples were collected from three groups of individuals who underwent cholecystectomy: 20 patients with non‐gallstone diseases (such as gallbladder polyps, gallbladder adenomyomatosis, and other conditions requiring cholecystectomy), 10 asymptomatic patients with gallstones but without diabetes mellitus, and 10 asymptomatic patients with gallstones and diabetes mellitus. During surgery, approximately 1 g of liver tissue was excised from the edge of the right hepatic lobe. Subjects were excluded if they had a history of any of the following: antibiotic use within three months prior to sample collection; other digestive tract–related diseases; allergic or autoimmune diseases or ongoing immunomodulatory therapy; neurological or neurodevelopmental disorders or chronic pain syndromes; obesity (defined as a BMI ≥ 30 kg/m^2^); metabolic syndrome or severe malnutrition; or malignant tumors or ongoing cancer treatment. After collection, all samples were immediately transported in liquid nitrogen and stored long‐term at −80°C.

### 2.9 Experimental Animals

Adult male C57BL/6J and ob/ob mice (6–8 weeks old, weighing 20 ± 2 g) were purchased from Jiangsu Jichu Yaokang Biotechnology Co., Ltd. The animals had free access to food and water and were housed under a 12‐hour light–dark cycle with a 7‐day acclimation period. A mouse model of gallstone disease was established using a cholesterol‐rich lithogenic diet. Mice were fed either a normal diet (ND; containing 0.02% cholesterol) or a lithogenic diet (LD; containing 1.25% cholesterol, 15% total fat, and 0.5% cholic acid) for four weeks [19]. Ob/ob mice were used to simulate hyperglycemic conditions, and cholelith formation was induced in these mice using the same lithogenic diet (DMLD). To achieve hepatic CXCR2 knockdown, mice received an intravenous injection of AAV‐PGK‐shCXCR2 (Applied Biological Materials Inc., ABM) at a dose of 1 × 10^11^ vg/100 µL. The pAAV‐PGK vector was used as a negative control. The shRNA sequence targeting CXCR2 was as follows: 5′‐GAAGATTTCTTCAGTGGAGAT‐3′. All experiments were performed 28 days after AAV injection. To inhibit NETs formation, mice were administered deoxyribonuclease I (DNase I, 15 000 U/kg; Roche) via intraperitoneal injection once daily for four weeks [20]. To induce NETs formation, mice were treated with phorbol 12‐myristate 13‐acetate (PMA, 200 µg/kg; Roche) via intraperitoneal injection once daily for four weeks [21]. To preserve hepatic tight junctions, mice were treated with SEW2871 (0.5 mg/kg) via intraperitoneal injection once daily for four weeks according to the manufacturer's instructions. To investigate the effect of sarcosine on cholelith formation, mice received sarcosine (400 mg/kg) via intraperitoneal injection once daily for four weeks as recommended by the manufacturer. To enhance CXCR2 activity, mice were treated with the CXCR2 agonist Ac‐PGP (Ac‐Pro‐Gly‐Pro‐OH, 10 mg/kg) via intraperitoneal injection once daily for four weeks [22]. To investigate the potential off‐target effects of sarcosine, mice were treated with the CXCR2 antagonist SB225002 (2 mg/kg, HY‐16711) via intraperitoneal injection once daily for four weeks, according to previously established protocols [23]. Mice in the control group received an equivalent volume of normal saline via intraperitoneal injection. SB225002, Ac‐PGP, SEW2871, and sarcosine were all obtained from MedChemExpress (MCE), Shanghai, China.

### 2.10 Cell Culture

The human immortalized hepatocyte cell line (THLE‐2) was obtained from Wuhan Procell Life Science & Technology Co., Ltd., and cultured in THLE‐2–specific medium (CM‐0833; Procell). The human promyelocytic leukemia cell line (HL‐60) was purchased from Shanghai Biowing Biotechnology Co., Ltd., and maintained in HL‐60–specific medium (CM‐0110; Procell). HL‐60 cells were induced to differentiate into neutrophil‐like cells by culturing with 1.25% dimethyl sulfoxide (DMSO; HY‐Y0320, MCE) for four days. To induce NETs formation, 10 mL of cells (1 × 106 cells/mL) were seeded into T75 flasks and treated with PMA (50 nm; P8139, Sigma‐Aldrich) for four hours, following previously described protocols [24].

For Transwell co‐culture experiments, THLE‐2 cells were seeded into the lower chamber of Transwell inserts, and DMSO‐induced neutrophil‐like cells derived from HL‐60 were seeded into the upper chamber. The control group consisted of THLE‐2 cells cultured alone. In the experimental groups, after induction of NETs formation by PMA, the lower chamber was supplemented with either the vehicle control (DMSO), the neutrophil elastase (NE) inhibitor sivelestat (0.05 µM; HY‐17443, MCE), the myeloperoxidase (MPO) inhibitor AZD5904 (140 nm; HY‐111341, MCE), the competitive matrix metalloproteinase 9 (MMP9) inhibitor SB‐3CT (600 nm; HY‐12354, MCE), or a combination of sivelestat and AZD5904, followed by 12 h of co‐culture. The degree of tight junction disruption in THLE‐2 cells was assessed by detecting the expression levels of tight junction‐associated proteins zonula occludens‐1 (ZO‐1), occludin, and claudin‐1.

### 2.11 Measurement of Transepithelial Electrical Resistance (TEER)

Electrodes were equilibrated in Hanks' Balanced Salt Solution pre‐warmed to 37°C for 20 min. Culture medium was aspirated from all wells and replaced with pre‐warmed HBSS, followed by an equilibration period. An appropriate volume of HBSS was then added to both the apical and basolateral chambers. TEER values were measured and normalized by subtracting the resistance value of blank inserts (without cells) [25].

### 2.12 Measurement of Gallstone Weight and Bile Collection

Mice were fasted for six hours but allowed free access to water. After euthanasia, the presence of gallstones was examined and photographed. The gallbladder was carefully removed intact, and all its contents were extracted and placed on a glass slide to allow complete evaporation of the liquid. The remaining solid residue was then weighed, and gallstone formation in the bile was examined using a polarized light microscope. Bile collection was performed according to a previously described method [19]. Briefly, mice were anesthetized with isoflurane and maintained at 37°C on a heating pad. A PE‐10 catheter was used to cannulate the common bile duct for bile collection. The volume of bile was measured every 30 min. Collected bile samples were immediately transported on cold nitrogen and stored long‐term at −80°C.

### 2.13 Immunofluorescence and Immunohistochemistry

Tissue paraffin blocks were sectioned, followed by dewaxing, dehydration, infiltration, and sealing. The sections were then incubated with primary antibodies against MPO (1:200, ab300650, Abcam), Ly6G (1:200, ab210204, Abcam), CXCR2 (1:150, A3301, Abclonal), CitH3 (1:200, ab281584, Abcam), ZO‐1 (1:500, 21773‐1‐AP, Proteintech), occluding (1:500, 27260‐1‐AP, Proteintech) and claudin‐1 (1:200, 28674‐1‐AP, Proteintech) at 4°C. The secondary antibodies used were CoraLite 488–conjugated goat anti‐rabbit IgG (H+L) (SA00013‐2) and CoraLite 594–conjugated goat anti‐mouse IgG (H+L) (SA00013‐3), both obtained from Proteintech. Immunofluorescence images were acquired using an LSM510 laser scanning confocal microscope (Carl Zeiss Microscopy, USA) equipped with an inverted optical system. Image acquisition and analysis were performed using Zen software (Carl Zeiss Microscopy, USA).

For immunohistochemical analysis, tissue preparation, staining, and mounting were carried out as described previously [26]. Paraffin‐embedded tissue sections were incubated with an anti‐CXCR2 primary antibody (1:150, A3301, Abclonal) overnight at 4°C. After washing with phosphate‐buffered saline (PBS), the sections were incubated with the appropriate secondary antibody at room temperature for one hour and counterstained with hematoxylin.

### 2.14 Western Blot (WB) Analysis

Cellular proteins were extracted and separated by sodium dodecyl sulfate–polyacrylamide gel electrophoresis. The separated proteins were then transferred onto nitrocellulose membranes, which were subsequently blocked to prevent nonspecific binding. The membranes were incubated with the appropriate primary antibodies, followed by horseradish peroxidase‐conjugated secondary antibodies. Protein bands were visualized using an enhanced chemiluminescence detection system. The primary antibodies used were CXCR2 (1:1,000, A3301, Abclonal), β‐actin (1:10,000, 20536‐1‐AP, Proteintech), CYP8B1 (1:3,000, 55217‐1‐AP, Proteintech), CYP27A1 (1:1,000, 14739‐1‐AP, Proteintech), CYP7A1 (1:3,000, 18054‐1‐AP, Proteintech), HMGCR (1:1,000, CSB‐PA010565LA01HU, CUSABIO), FXR (1:5,000, 25055‐1‐AP, Proteintech), ABCG5 (1:1,000, TD8401S, Abmart), ABCG8 (1:1,000, A1880, Abclonal).

### 2.15 RT‐qPCR Detection

Total RNA was extracted from cells using TRIzol reagent (Ambion, USA). RNA concentration and purity were assessed using a NanoDrop 2000c spectrophotometer (Thermo Scientific, USA) [27]. According to the manufacturer's protocol, 2 µg of total RNA was reverse transcribed into complementary DNA (cDNA) using the Transcriptor First‐Strand cDNA Synthesis Kit (Roche, Germany). β‐actin was used as the internal reference gene. Quantitative real‐time PCR was performed with SYBR Green Master Mix (ROX) (Roche, Germany) on a 7500 Fast Real‐Time PCR System (Applied Biosystems, USA) following the manufacturer's instructions. Relative gene expression was calculated using the 2^−(ΔΔ^ *^Ct^* ^)^ method. The primer sequences were as follows:

- OCLN (occludin): forward, 5′‐TGGCAAGCGATCATACCCAGAG‐3′; reverse, 5′‐CTGCCTGAAGTCATCCACACTC‐3′.
- TJP1 (tight junction protein 1): forward, 5′‐GTTGGTACGGTGCCCTGAAAGA‐3′; reverse, 5′‐GCTGACAGGTAGGACAGACGAT‐3′.
- CLDN1 (claudin‐1): forward, 5′‐GGACTGTGGATGTCCTGCGTTT‐3′; reverse, 5′‐GCCAATTACCATCAAGGCTCGG‐3′.
- CXCR2: forward, 5′‐CTCTATTCTGCCAGATGCTGTCC‐3′; reverse, 5′‐ACAAGGCTCAGCAGAGTCACCA‐3′.

### 2.16 Elisa

The C‐X‐C chemokine receptor type 2 (CXCR2) enzyme‐linked immunosorbent assay (ELISA) kit (LP‐030858) was obtained from Shanghai LUPUKEJI. The MPO ELISA kit (A044‐1‐1) was purchased from Nanjing Jiancheng Bioengineering Institute. The human citrullinated histone H3 (CitH3) ELISA kit (JL48343) and the human NE ELISA kit (JL54592D) were obtained from Shanghai Jianglai Biotechnology. Human and mouse MPO–DNA complex detection kits (BP04025 and BP00283, respectively) were purchased from Shanghai Baipeng Biotechnology Co., Ltd., and assays were performed according to the manufacturer's instructions. Total cholesterol (A111‐1‐1) and total bile acid (E003‐2‐1) levels were measured using kits from Nanjing Jiancheng Bioengineering Institute, following the manufacturer's protocols. Phospholipid concentration was quantified using a Wako kit (296‐63801, Osaka, Japan) in accordance with the manufacturer's instructions. Absorbance was measured using a BioTek Synergy NEO multimode microplate reader (BioTek Instruments, USA).

### 2.17 Immunogold Labeling Electron Microscopy

Liver tissues were fixed in a mixture of 4% paraformaldehyde and 0.1% glutaraldehyde, followed by dehydration through a graded ethanol series and embedding in epoxy resin. Ultrathin sections (60–80 nm) were cut using an ultramicrotome. After blocking with 1% BSA, the sections were incubated with a mixed primary antibody solution (MPO and citH3, diluted 1:100) at 4°C overnight. Following PBS washes, the sections were treated with a mixture of 10 nm colloidal gold‐conjugated secondary antibodies for 1 h at room temperature. After thorough rinsing, double staining was performed with uranyl acetate and lead citrate. Finally, the samples were observed and imaged under a Hitachi HT7800 transmission electron microscope (Japan).

### 2.18 Bulk RNA Data Analysis

After normalization, annotation, and cleaning of the clinical data from the GSE23343 dataset in the Gene Expression Omnibus (GEO) database, the limma package in R was used to identify differentially expressed genes (DEGs) between 10 patients with type 2 diabetes mellitus and 7 subjects with normal glucose tolerance [28, 29]. Genes with a *p‐*value less than 0.05 were considered significantly differentially expressed.

### 2.19 Single‐Cell RNA Sequencing (scRNA‐Seq) Cell Preparation

Immediately after tissue collection, samples were pre‐cooled in ice‐cold PBS (Hyclone, SH30256.01) and dissociated using the SeekGene Tissue Dissociation Kit A Pro (SeekGene, K01801‐30) according to the manufacturer's protocol. DNase I (Sigma, 9003‐98‐9) was added as needed, depending on the viscosity of the tissue homogenate. Following red blood cell removal using red blood cell lysis buffer (Solarbio, R1010), cell count and viability were assessed with the SeekMate Tinitan fluorescence cell counter (SeekGene, M002C) using acridine orange/propidium iodide (AO/PI) staining. Based on the viability and debris ratio, fragments and dead cells were selectively removed using Miltenyi debris and dead cell removal kits (130‐109‐398 and 130‐090‐101, respectively). Finally, viable cells were washed twice with RPMI 1640 medium (Gibco, 11875119) and resuspended in RPMI 1640 medium containing 2% fetal bovine serum (Gibco, 10100147C), adjusting the final concentration to 1 × 10^6^ cells/mL.

### 2.20 scRNA‐Seq Library Construction and Sequencing

The single‐cell RNA sequencing library was prepared using the SeekOne DD Single‐Cell 5′ End Library Construction Kit (SeekGene, K00501). The workflow was as follows: an appropriate number of cells was mixed with reverse transcription reagents and loaded into the sample wells of the SeekOne DD Chip S3. Gel beads and partitioning oil were then added to the corresponding wells of the chip. After emulsification, a reverse transcription reaction was carried out at 42°C for 90 min, followed by enzyme inactivation at 85°C for 5 min. The cDNA was subsequently purified from the emulsified droplets and subjected to PCR amplification. The amplified cDNA products were used to construct the gene expression sequencing library. The amplified products were fragmented, end‐repaired, A‐tailed, and ligated with sequencing adapters. Index PCR was then performed to amplify DNA fragments containing both cell barcodes and unique molecular identifiers (UMIs). The indexed library was purified using VAHTS DNA Clean Beads (Vazyme, N411‐01), and quality control was performed with a Qubit fluorescence spectrometer (Thermo Fisher Scientific, Q33226) and a Bio‐Fragment Analyzer (Bioptic, Qsep400). Paired‐end sequencing (2 × 150 bp) was conducted on the Illumina NovaSeq X Plus platform.

### 2.21 Bioinformatics Analysis of scRNA‐Seq

Single‐cell RNA sequencing data (GSE239612) were obtained from the GEO database (https://www.ncbi.nlm.nih.gov/geo) and analyzed using the Seurat R package (version 4.4.0) [30, 31]. Genes expressed in fewer than three cells and cells expressing fewer than 200 genes were excluded. Cell quality control was performed based on the number of expressed genes per cell and the percentage of mitochondrial gene expression. Cells with more than 6000 detected genes or a mitochondrial gene percentage exceeding 5% were removed. Data normalization was performed using the SCTransform method, and dimensionality reduction was conducted via principal component analysis. Cells exhibiting similar expression profiles were clustered using the FindNeighbors and FindClusters functions. Visualization was achieved using uniform manifold approximation and projection (UMAP). The FindAllMarkers function was applied to identify DEGs, and the top ten genes with the highest log2 fold change within each cluster were defined as marker genes. Cell type annotation was conducted by integrating canonical cell markers with the marker genes from each cluster, referencing the PanglaoDB database (https://panglaodb.seindex.html) and CellMarker [32, 33].

For self‐generated sequencing data, raw reads were quality‐controlled using the Fastp tool, including adapter trimming and removal of low‐quality bases [34]. Deduplication was then performed to extract cell barcode information, followed by alignment of the filtered reads to the human GRCh38 reference genome using the Seeksoul analysis platform (SeekGene, version 1.2.1). Finally, a feature‐barcode matrix was generated for downstream analyses, such as creating a Seurat object in R for subsequent single‐cell analysis.

### 2.22 Statistical Analysis

All analyses of the NHANES database were performed in accordance with the guidelines of the NHANES (Centers for Disease Control and Prevention/National Center for Health Statistics, 2018), accounting for the complex sampling design by applying appropriate subsample weights, stratification, and primary sampling units. For continuous variables following a normal or approximately normal distribution, data were expressed as the mean ± standard error. Survey‐weighted univariate and multivariate logistic regression analyses were conducted to identify potential risk factors for gallstone disease. All statistical analyses were performed using the “nhanesR” and “survey” packages in R software (version 4.2.1https://www.r‐project.org;)

For other quantitative data, results were presented as the mean ± standard deviation. The Student's *t‐*test was used for comparisons between two groups, and one‐way analysis of variance (ANOVA) was employed for comparisons among three or more groups. A *P*‐value < 0.05 was considered statistically significant and denoted by one asterisk. *P* < 0.01 by two asterisks, and *P* < 0.001 by three asterisks.

## 3 Results

### 3.1 Analysis of the NHANES Database Indicates That Diabetes Is a Risk Factor for Gallstone Formation

A total of 3972 participants were included in this study. The baseline characteristics of the participants are presented in Table S1. Among them, 366 individuals had gallstones, yielding an incidence rate of 9.21%. Univariate logistic regression analysis revealed that diabetes was a significant risk factor for gallstone formation (*P* < 0.001). Moreover, diabetes‐related indicators—including fasting plasma glucose (FPG), fasting serum insulin (FSI), glycated hemoglobin (HbA1c), METS‐IR, and HOMA‐IR—were also positively associated with gallstones (all *P* < 0.05) (Table S2). To further assess the relationship between diabetes and gallstone formation, logistic regression analyses were performed. As shown in Table 1, across the unadjusted model, the partially adjusted models (Model 1 and Model 2), and the fully adjusted model (Model 3), diabetes consistently remained associated with an increased risk of gallstones (*P* < 0.05). The diabetes‐related indicator FSI served as a significant risk factor in the partially adjusted model, while FPG, HbA1c, HOMA‐IR, and METS‐IR were positively correlated with gallstone occurrence in all models. The fully adjusted model accounted for multiple confounding variables, including age, sex, race, smoking status, alcohol consumption, poverty ratio, and BMI. Collectively, these findings indicate that diabetes is a significant risk factor for gallstone formation.

**TABLE 1:** The relationship between Diabetes Mellitus and gallstone.

	Crude model		Model 1		Model 2		Model 3	
character	OR(95%CI)	*P‐*value	OR (95%CI)	*P‐*value	OR (95%CI)	*P‐*value	OR (95%CI)	*P‐*value
DM	2.26 (1.68–3.04)	<0.0001	1.92 (1.47–2.50)	<0.0001	1.98 (1.52–2.59)	<0.0001	1.60 (1.17–2.19)	0.005
FPG	1.10 (1.04–1.16)	<0.001	1.11 (1.05–1.18)	<0.001	1.12 (1.06–1.18)	<0.001	1.09 (1.03–1.15)	0.01
FSI	1.01 (1–1.02)	0.03	1.01 (1–1.03)	0.04	1.01 (1–1.03)	0.05	1.01 (1–1.02)	0.12
HbA1c	1.28 (1.18–1.40)	<0.0001	1.26 (1.12–1.42)	<0.001	1.28 (1.15–1.43)	<0.0001	1.20 (1.05–1.37)	0.01
HOMA‐IR	1.03 (1.01–1.05)	0.002	1.03 (1.01–1.06)	0.005	1.03 (1.01–1.06)	0.01	1.03 (1.01–1.05)	0.01
METS‐IR	1.03 (1.02–1.04)	<0.0001	1.04 (1.03–1.04)	<0.0001	1.04 (1.03–1.04)	<0.0001	1.04 (1.03–1.05)	<0.0001
SHR	0.63 (0.17–2.32)	0.48	1.65 (0.44–6.12)	0.44	1.65 (0.45–6.11)	0.44	1.30 (0.39–4.36)	0.66

DM: Diabetes Mellitus; FPG: Fasting Plasma Glucose; FSI: Fasting Serum Insulin; HbA1c: Hemoglobin A1c; HOMA‐IR: Homeostasis Model Assessment of Insulin Resistance; METS‐IR: Metabolic Score for Insulin Resistance; SHR: Stress Hyperglycemia Ratio.

Model 1: Adjusted for age, sex, race.

Model 2: Adjusted for age, sex, race, smoking status, and alcohol.

Model 3:Adjusted for age, sex, race smoking status, alcohol consumption, poverty ratio, and BMI.

### 3.2 Mendelian Randomization Analysis and Animal Experiments Confirm the Causal Relationship Between Diabetes and Gallstone Formation

**FIGURE 2:**
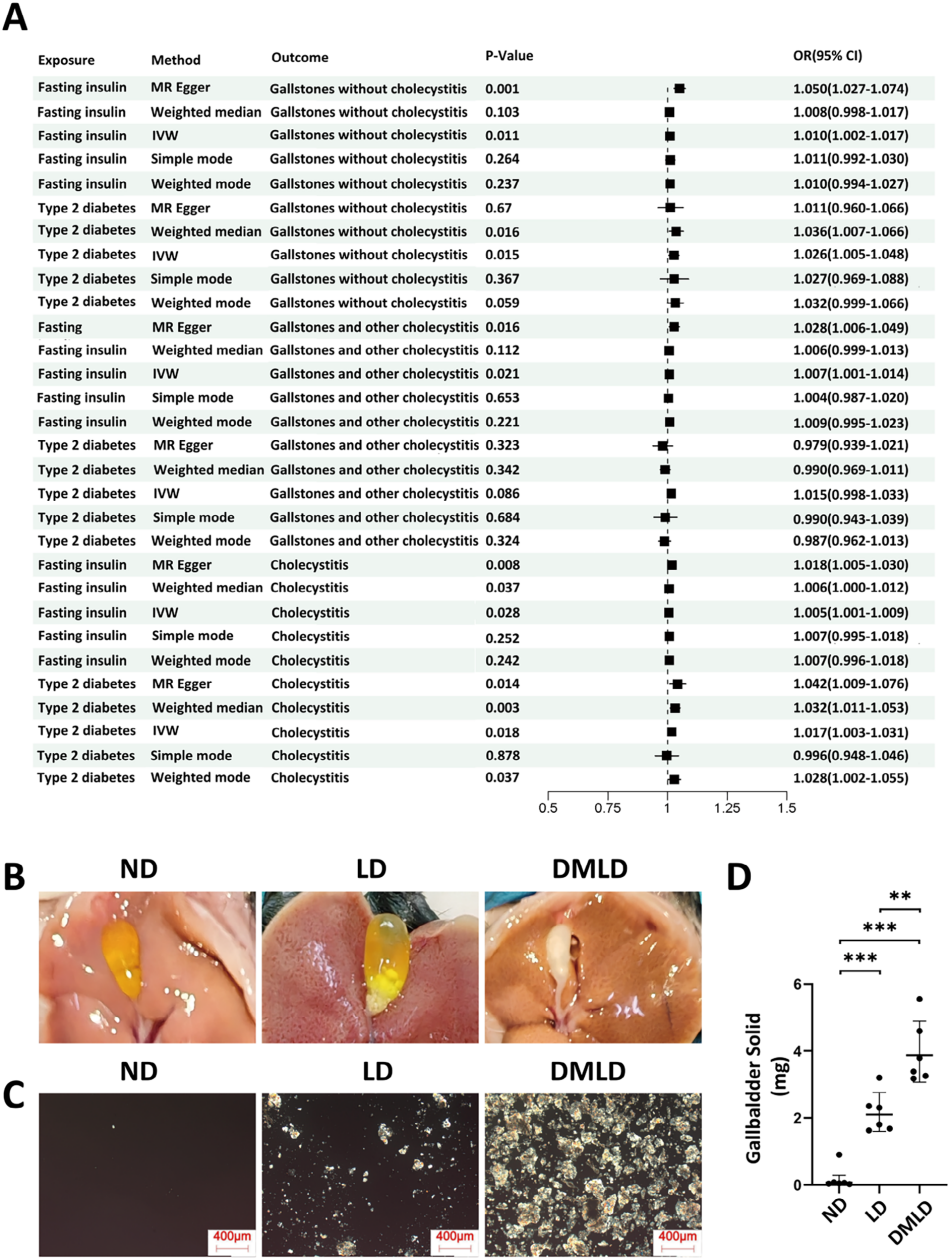
The Mendelian randomization analysis demonstrated a causal relationship between diabetes and gallstone formation, and animal experiments further confirmed that diabetes promotes gallstone formation in mice. (A) Mendelian randomization analysis of diabetes and gallstone; (B) representative images of the gallbladders of mice fed a regular diet (ND), lithogenic diet (LD), or diet‐induced diabetes diet (DMLD) (*n* = 6 per group); (C) polarized light microscopy images of bile from the gallbladders of each mouse group (*n* = 6 per group); (D) gallstone weights in the gallbladders of each group (*n* = 6 per group). Student's *t*‐test was used for comparisons between two groups, and one‐way ANOVA was used for comparisons among three or more groups. *P < 0.05 was considered statistically significant, **P < 0.01, ***P < 0.001.

MR analysis was employed to investigate the causal association between diabetes‐related phenotypes and gallstone formation. As shown in Figure 2A, the results from five MR analysis methods are presented. The analysis revealed that fasting insulin levels and type 2 diabetes were significantly and positively associated with the risk of gallstones. Using the IVW method to assess exposure factors and various gallstone outcomes, type 2 diabetes showed a significant positive association with gallstones without cholecystitis, gallstones accompanied by other cholecystitis, and cholecystitis alone. These findings suggest that type 2 diabetes may increase the risk of both gallstones and cholecystitis. Furthermore, fasting blood glucose remained significantly and positively correlated with all three gallstone‐related outcomes, indicating that elevated serum insulin levels in patients with type 2 diabetes may play a key mechanistic role in gallstone formation. To further validate the causal link between diabetes and gallstone formation, animal experiments were conducted using ob/ob mice to simulate diabetes. As shown in Figure 2B–D, compared with mice fed a normal diet (ND), those fed a lithogenic diet (LD) exhibited increased gallstone formation, while DMLD showed a further significant increase in gallstone formation.

### 3.3 Diabetes Promotes an Increase in CXCR2 Expression

**FIGURE 3:**
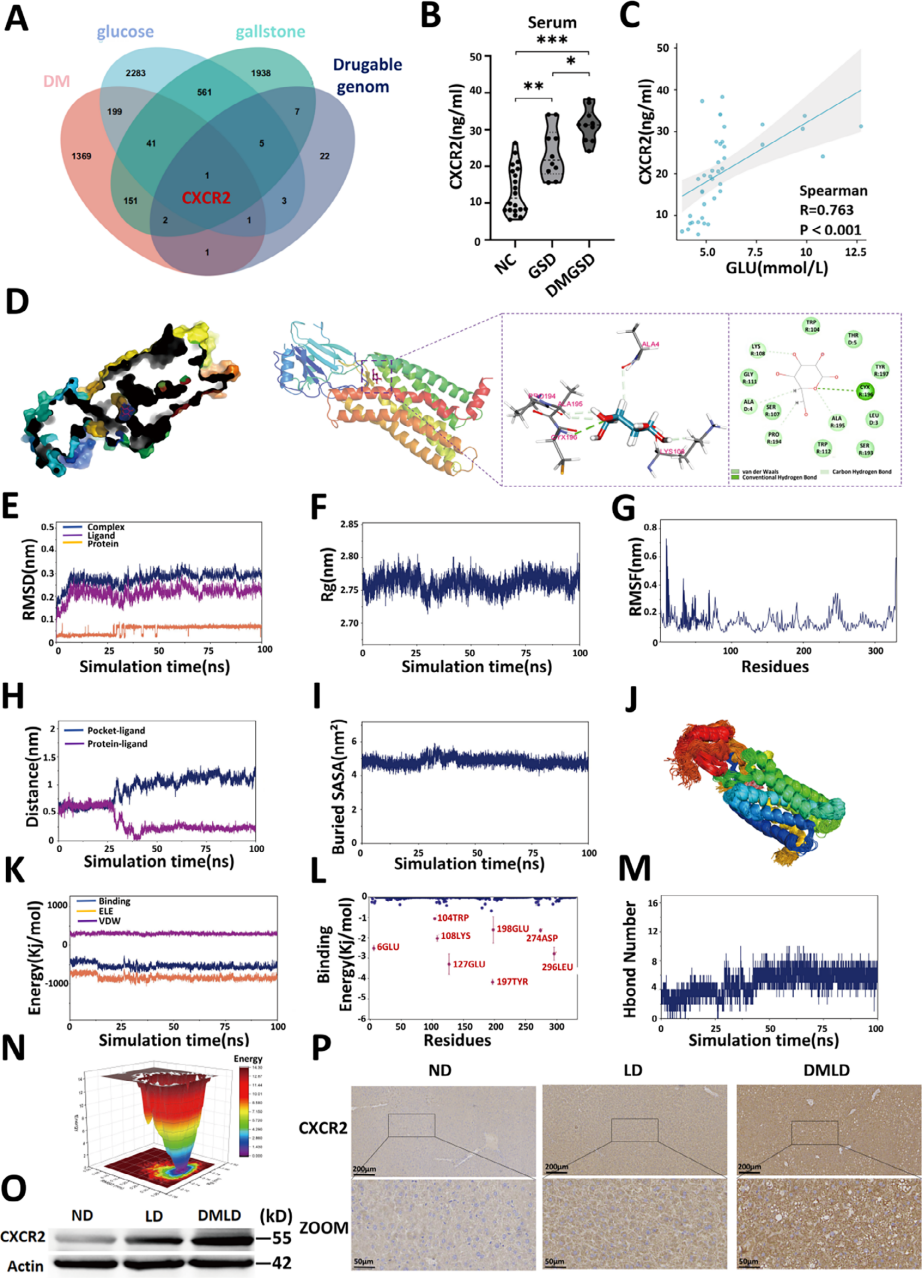
Glucose binding to CXCR2 promotes increased CXCR2 expression. (A) combined analysis of diabetes‐ and gallstone‐related genes across multiple databases and identification of overlapping genes; (B) serum CXCR2 levels in the control group (NC), gallstone group (GSD), and diabetes with gallstone group(DMGSD) (*n* = 20 NC group, *n* = 10 GSD and DMGSD group); (C) correlation analysis between human serum glucose levels and CXCR2 levels (*n *= 40); (D) interaction between CXCR2 protein and glucose; (E) RMSD of the complex, CXCR2 protein, and glucose ligand; (F) Rg of the complex; (G) RMSF of CXCR2 protein within the complex; (H) distance between the CXCR2 protein binding site and glucose (Dock site–ligand); (I) buried SASA between glucose and CXCR2 protein; (J) superimposed simulated conformations; (K) binding energy components VDW and ELE between glucose and CXCR2 protein; (L) amino acid binding energy contributions; (M) number of hydrogen bonds (Hbond number); (N) FEL free energy landscape; (O) western blotting maps of CXCR2 protein in the livers of mice fed a normal diet (ND), lithogenic diet (LD), or diabetes with lithogenic diet(DMLD) (*n* = 6 per group); (P) immunohistochemical staining of liver tissues from each group (*n* = 6 per group). Student's *t*‐test was used for comparisons between two groups, and one‐way ANOVA was used for comparisons among three or more groups. *P < 0.05 was considered statistically significant, **P < 0.01, ***P < 0.001.

To elucidate the specific mechanism through which diabetes promotes gallstone formation, we analyzed DEGs in the livers of patients with diabetes and those with normal glucose tolerance using the GEO dataset GSE23343. A total of 1765 genes exhibited significant differential expression. To predict potential glucose targets, we utilized three databases—TargetNet, Swiss Target Prediction, and SEA—and identified 3094 genes potentially targeted by glucose. Additionally, using the GeneCards database, we identified 2706 candidate pathogenic genes associated with gallstones. Mendelian randomization analysis of gallstone‐related druggable genes in the FinnGen database identified 42 druggable genes associated with gallstone disease. Comparative analysis revealed that CXCR2 was the common overlapping gene among these datasets (Figure 3A). Subsequent Mendelian randomization validation confirmed that CXCR2 is causally related to gallstone disease, and the causal inference was statistically robust (Figure S2A–D). Measurement of CXCR2 levels in serum samples from the control group (NC), gallstone disease group (GSD), and diabetes‐associated gallstone disease group (DMGSD) showed a significant increase in CXCR2 concentration in the DMGSD group compared with the other two groups (Figure 3B). Furthermore, correlation analysis demonstrated a positive association between serum CXCR2 levels and blood glucose concentration (Figure 3C). Collectively, these results suggest that CXCR2 may play a crucial role in mediating the promotive effect of diabetes on gallstone formation.

We performed molecular docking and MD analyses to investigate the interaction between glucose and the CXCR2 protein. The results indicated that the amino acid residue CYX‐196 in CXCR2 formed hydrogen bonds with glucose, while residues such as TRP‐104, THR‐197, and TRP‐112 engaged in van der Waals interactions with the small molecule (Figure 3D). Glucose demonstrated stable binding to CXCR2, and the hydrogen bonds became increasingly stable over time. Both the binding energy and binding affinity exhibited strong values, suggesting a robust interaction between glucose and CXCR2. Energy decomposition analysis revealed that electrostatic interactions contributed most significantly to the total binding energy, van der Waals forces played a secondary role, and hydrophobic interactions provided additional stabilizing effects (Figure 3E–N). To further confirm the direct binding between glucose and the CXCR2 protein, SPR assays were performed. SPR analysis demonstrated a high‐affinity interaction and concentration‐dependent binding of glucose to CXCR2. (Figure S3A,B). Moreover, we examined CXCR2 protein expression in the liver tissues of mice from the ND, LD, and DMLD groups. Compared with the ND group, CXCR2 expression in the LD group was elevated, and expression in the DMLD group was further and significantly upregulated (Figure 3O,P).

### 3.4 Increased Expression of CXCR2 in Liver Neutrophils Is a Key Factor in Diabetes‐Induced Gallstone Formation

**FIGURE 4:**
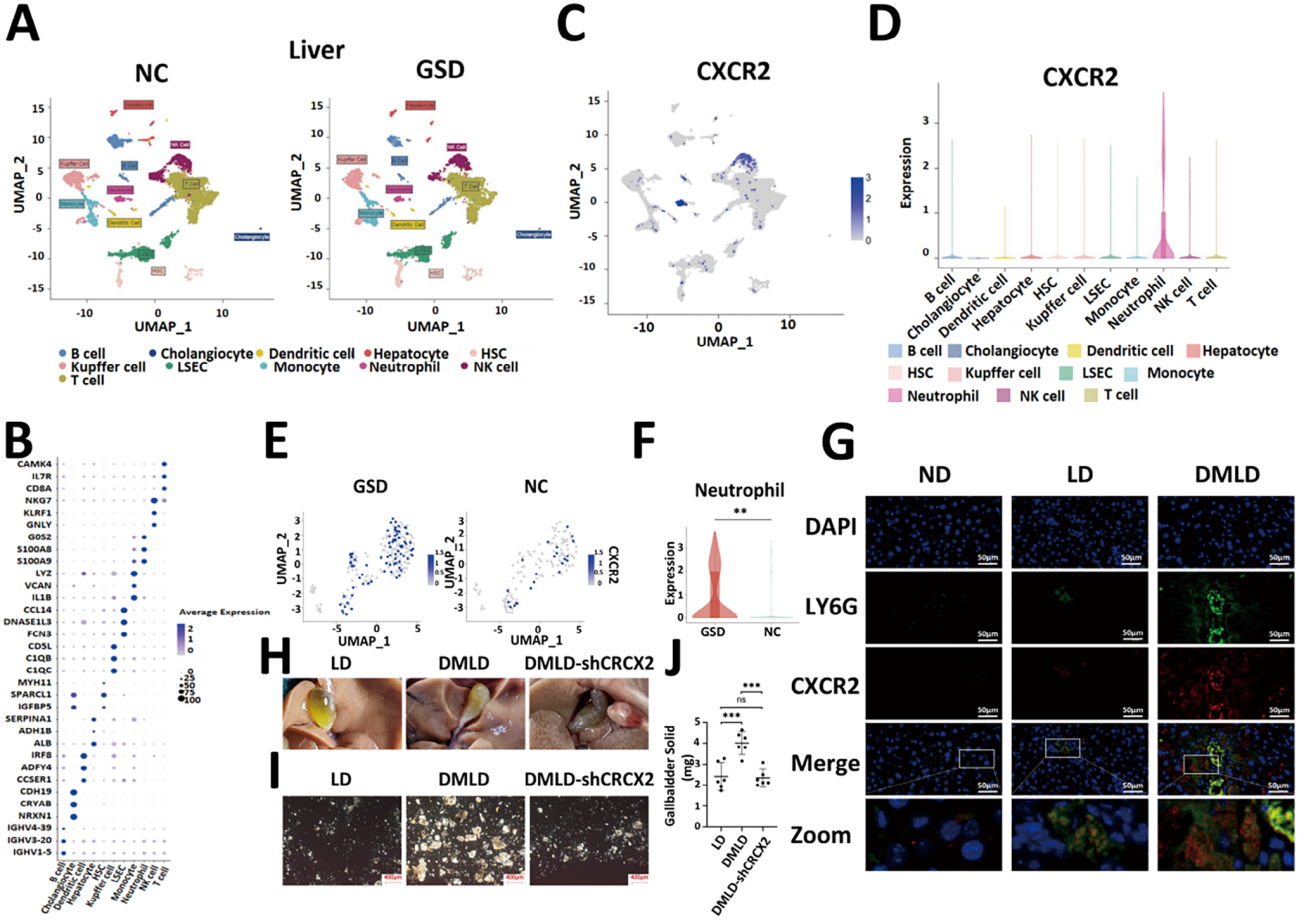
The increased expression of CXCR2 in liver neutrophils is a key factor in diabetes‐induced gallstone formation. (A) Liver tissue cell profiles of normal control group and patients with gallstones; (B) marker genes used to differentiate various cell types; (C) expression distribution of the CXCR2 gene; (D) expression pattern of the CXCR2 gene across different cell types; (E) UMAP visualization of CXCR2 gene expression in liver neutrophils from patients with gallstones and normal control group; (F) comparison of CXCR2 expression levels in liver neutrophils between patients with gallstones and normal control group; (G) dual immunofluorescence staining of LY6G and CXCR2 in liver tissues of mice fed a normal diet(ND), lithogenic diet(LD), or diabetes‐induced lithogenic diet(DMLD) (*n* = 6 per group); (H) representative images of gallbladders from mice fed a lithogenic diet, diabetes‐induced lithogenic diet, or diabetes‐induced lithogenic diet with CXCR2 gene interference (*n* = 6 per group); (I) polarized light microscopy images of bile in the gallbladders of mice from each group (*n* = 6 per group); (J) gallstone weights in the gallbladders of mice from each group (*n* = 6 per group). Student's *t*‐test was used for comparisons between two groups, and one‐way ANOVA was used for comparisons among three or more groups. *P < 0.05 was considered statistically significant, **P < 0.01, ***P < 0.001.

Single‐cell transcriptomic sequencing was performed on liver tissues from patients with GSD and from healthy individuals without gallstones (NC group). Each group included one sample, yielding a total of 17 310 cells. After quality control, 13 392 high‐quality cells were retained. The number of detected genes, UMI counts, and the proportion of mitochondrial genes in each cell were all within normal ranges. Subsequently, clustering analysis of gene expression profiles identified 11 distinct cell types. Each cell cluster was annotated based on the expression of known marker genes (Figure 4A,B) and visualized using UMAP (Figure 4C). CXCR2 expression was predominantly observed in neutrophils (Figure 4D). Further subpopulation analysis of hepatic neutrophils revealed that CXCR2 expression in the liver neutrophils of the GSD group was markedly higher than that in the NC group (Figure 4E,F).

We performed dual immunofluorescence staining for CXCR2 and LY6G in liver tissues from mice in the ND, LD, and DMLD groups. Co‐localization analysis revealed that CXCR2 and LY6G were co‐expressed, and CXCR2 expression was significantly elevated in diabetic mice (Figure 4G). These findings further confirmed that diabetes enhances CXCR2 expression in mouse liver tissues and that CXCR2 is primarily localized in neutrophils. To investigate the role of CXCR2 in diabetes‐mediated gallstone formation, an adeno‐associated viral vector carrying CXCR2 shRNA (AAV‐PGK‐shCXCR2) was injected into the tail vein of diabetic mice to suppress hepatic CXCR2 expression. Diabetic mice injected with the control vector (AAV‐PGK) served as the negative control (Figure S3A,B). All mice were then fed a lithogenic diet. The results demonstrated that diabetic mice injected with the control vector exhibited markedly increased gallstone formation compared with normal mice, whereas CXCR2 inhibition in the liver completely abolished the diabetes‐induced promotion of gallstone formation (Figure 4H–J). These results indicate that diabetes promotes gallstone formation through CXCR2 expressed in hepatic neutrophils.

### 3.5 NETs Play a Significant Role in Gallstone Disease

**FIGURE 5:**
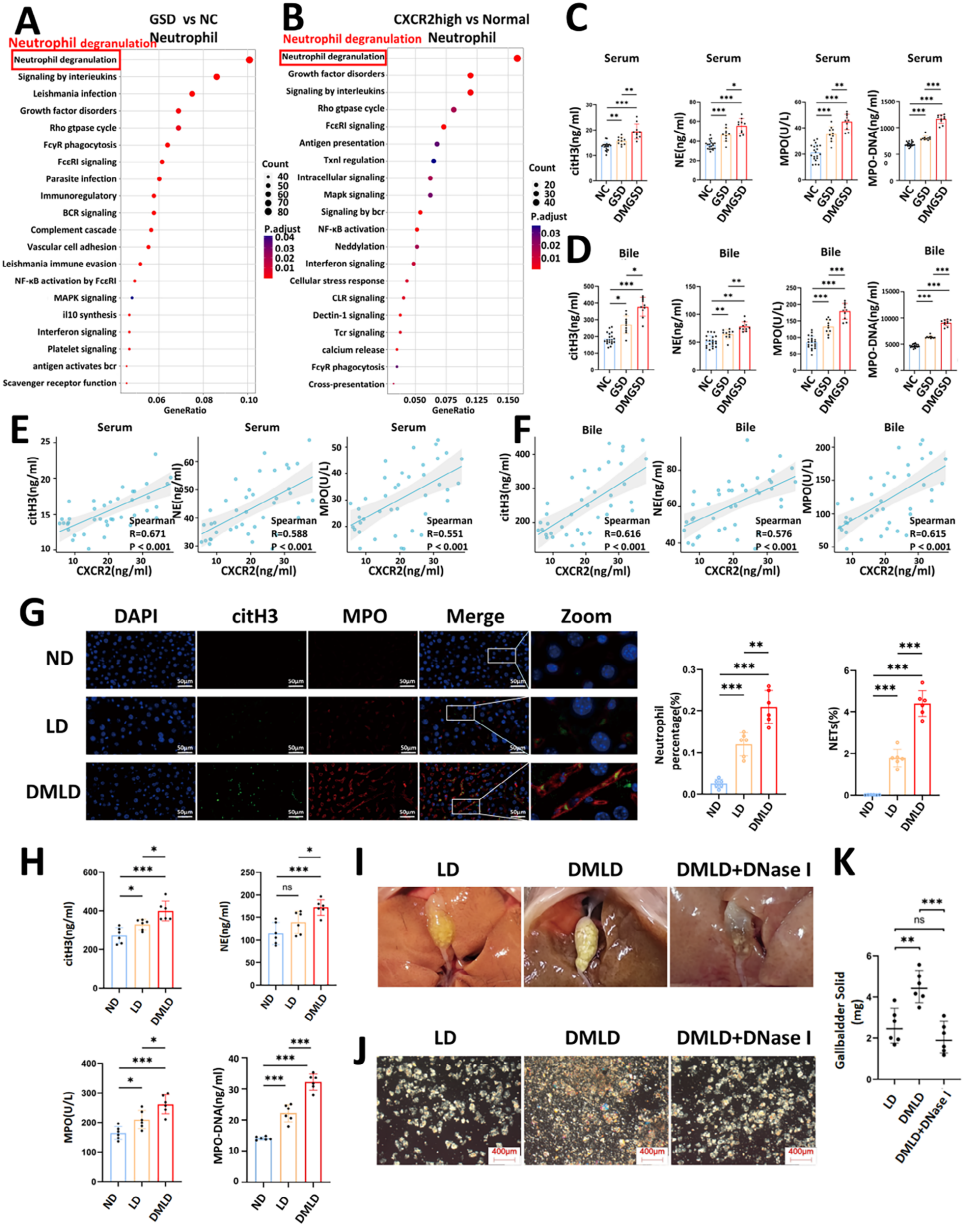
NETs play a significant role in gallstone disease. (A) Reactome enrichment analysis of differentially expressed genes in neutrophils from normal control group and patients with gallstones; (B) Reactome enrichment analysis of differentially expressed genes in neutrophils with high versus low CXCR2 expression in human liver tissues; (C) levels of citH3, NE, MPO and MPO‐DNA in the serum of normal control group (NC), patients with gallstones (GSD), and patients with diabetes‐related gallstones (DMGSD) (*n* = 20 NC group, *n *= 10 GSD and DMGSD group); (D) levels of citH3, NE, MPO and MPO‐DNA in bile from each group (*n* = 20 NC group, *n* = 10 GSD and DMGSD group); (E) correlation analysis between serum CXCR2 levels and serum levels of citH3, NE, and MPO (*n* = 40); (F) correlation analysis between serum CXCR2 levels and levels of citH3, NE, and MPO in bile (*n* = 40); (G) percentage of neutrophils and immunofluorescence images of NETs (MPO, red; citH3, green; the network structure represents NETs) in liver tissues of each group (*n* = 6 per group); (H) levels of citH3, NE, MPO and MPO‐DNA in bile from each group of mice (*n* = 6 per group); (I) representative images of gallbladders from mice fed a lithogenic diet(LD), diabetes‐related lithogenic diet (DMLD), and diabetes‐related lithogenic diet with DNase I treatment(DMLD+DNase I) (*n* = 6 per group); (J) polarized light microscopy images of bile in the gallbladders of each group (*n* = 6 per group); (K) gallstone weights in the gallbladders of each group (*n* = 6 per group). Student's *t‐*test was used for comparisons between two groups, and one‐way ANOVA was used for comparisons among three or more groups. *P < 0.05 was considered statistically significant, **P < 0.01, ***P < 0.001.

We first performed a Reactome enrichment analysis of the DEGs in neutrophils between the GSD group and the NC group. The DEGs were primarily enriched in the neutrophil degranulation signaling pathway (Figure 5A). Next, neutrophils with high CXCR2 expression were classified as CXCR2^high^, and Reactome enrichment analysis was performed to identify DEGs between CXCR2^high^ and CXCR2^low^ neutrophils. These DEGs were likewise enriched in the neutrophil degranulation signaling pathway (Figure 5B). Because neutrophil degranulation is a critical step in the formation of NETs, we hypothesized that CXCR2 may promote gallstone formation by facilitating NETs generation during the disease process [35]. CitH3, NE, MPO, and MPO‐DNA were used as molecular markers of NETs [36]. To further investigate the relationship between diabetes‐associated gallstone disease and NETs, we performed NETs staining on human liver tissues from the NC, GSD, and DNGSD groups. The levels of CitH3, NE, MPO, and MPO‐DNA were also measured in peripheral blood and bile samples from each group. The results demonstrated that NETs formation in liver tissues was markedly increased in gallstone patients with diabetes (Figure S5A), and that the levels of CitH3, NE, MPO, and MPO‐DNA in blood were significantly elevated (Figure 5C). Similarly, the concentrations of CitH3, NE, MPO, and MPO‐DNA in bile were substantially higher in gallstone patients complicated by diabetes (Figure 5D). Correlation analysis revealed that circulating CXCR2 levels were significantly and positively correlated with blood CitH3, NE, MPO, and MPO‐DNA concentrations (Figure 5E; Figure S5B), as well as with their levels in bile (Figure 5F; Figure S5C). We also examined NETs in gallbladder tissues from both human subjects and mouse models. Our results showed a significant increase in NETs formation and neutrophil infiltration in the gallstone group compared to the control group. However, no statistically significant difference was observed between the gallstone group and the group with diabetes complicated by gallstones (Figure S5D,E). Because gallstone assembly fundamentally depends on the presence of NETs, we speculate that diabetes may promote NETs formation in the liver through CXCR2 activation, thereby accelerating gallstone development [37].

We further evaluated NETs formation in mice fed an ND, LD, and DMLD. The number of neutrophils and the accumulation of NETs in the livers of DMLD mice were markedly increased (Figure 5G). Moreover, CitH3, NE, MPO, and MPO‐DNA levels in bile were significantly elevated in DMLD mice (Figure 5H). Intraperitoneal administration of DNase I effectively suppressed the diabetes‐induced enhancement of gallstone formation in mice (Figure 5I–K).

### 3.6 CXCR2 Promotes Gallstone Formation by Inducing the NETs Formation

**FIGURE 6:**
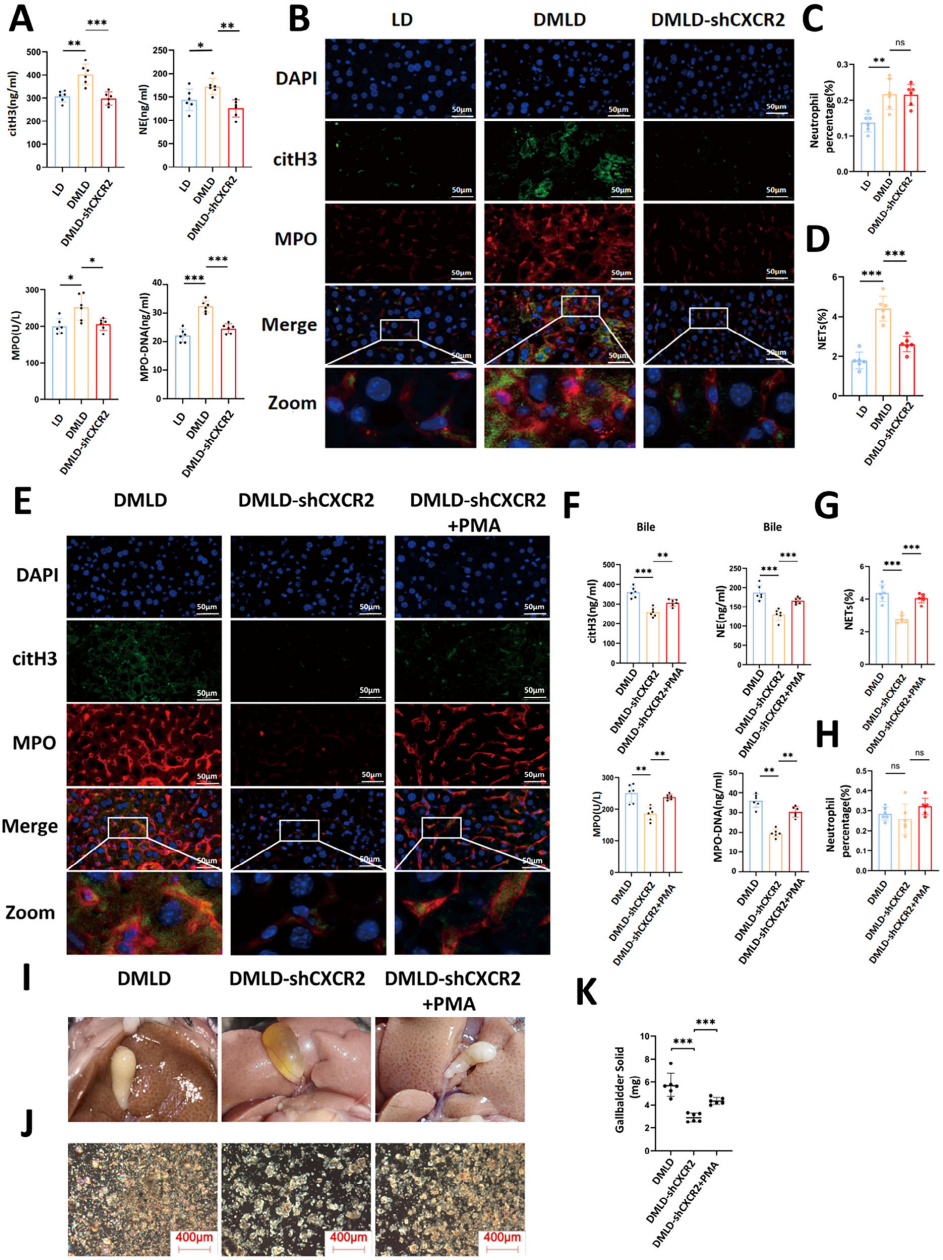
CXCR2 promotes gallstone formation by inducing the formation of NETs. (A) Levels of citH3, NE, MPO and MPO‐DNA in bile from mice fed a lithogenic diet(LD), diabetes‐induced lithogenic diet (DMLD) and diabetes‐induced lithogenic diet with CXCR2 gene interference (*n* = 6 per group); (B) immunofluorescence images of NETs in liver tissues of each group (MPO, red; citH3, green; the network structure represents NETs) (*n* = 6 per group); (C) percentage of neutrophils in liver tissues from each group (*n* = 6 per group); (D) percentage of NETs in liver tissues from each group (*n* = 6 per group); (E) immunofluorescence images of NETs in liver tissues from mice fed a diabetes‐induced lithogenic diet (DMLD), diabetes‐induced lithogenic diet with CXCR2 gene interference (DMLD‐shCXCR2), and diabetes‐induced lithogenic diet with CXCR2 gene interference plus intraperitoneal injection of PMA (DMLD‐shCXCR2+PMA) (MPO, red; citH3, green; the network structure represents NETs) (*n *= 6 per group); (F) levels of citH3, NE, MPO and MPO‐DNA in bile from each group of mice (*n* = 6 per group); (G) percentage of NETs in liver tissues from each group (*n* = 6 per group); (H) percentage of neutrophils in liver tissues from each group (*n* = 6 per group); (I) representative images of gallbladders from each group of mice (*n* = 6 per group); (J) polarized light microscopy images of bile in the gallbladders of each group (*n* = 6 per group); (K) gallstone weights in the gallbladders of each group (*n* = 6 per group); Student's *t*‐test was used for comparisons between two groups, and one‐way ANOVA was used for comparisons among three or more groups. *P < 0.05 was considered statistically significant, **P < 0.01, ***P < 0.001.

We selected mice from the LD, DMLD, and DMLD with DMLD‐shCXCR2 groups to assess NETs‐related markers. The concentrations of CitH3, NE, and MPO in the bile of DMLD‐shCXCR2 mice were markedly decreased (Figure 6A), indicating that suppression of CXCR2 expression attenuated the diabetes‐induced enhancement of NETs formation in mouse bile. Additionally, immunofluorescence staining of liver tissues from each group revealed that the number of neutrophils and the accumulation of NETs in the livers of DMLD‐shCXCR2 mice were significantly reduced (Figure 6B–D), suggesting that inhibition of hepatic CXCR2 expression suppresses diabetes‐induced NETs formation in the liver. PMA, a classical inducer of NETs formation [38], was subsequently used in a rescue experiment. Intraperitoneal injection of PMA into DMLD‐shCXCR2 mice markedly reversed the inhibitory effect of CXCR2 knockdown on NETs formation (Figure 6E–H). PMA administration also reversed the suppressive effect of CXCR2 inhibition on gallstone formation in DMLD mice (Figure 6I–K). Collectively, these results demonstrate that diabetes promotes NETs formation through CXCR2 activation in neutrophils, which subsequently facilitates gallstone formation.

### 3.7 Diabetes Promotes the Release of NETs Into Bile by Disrupting Hepatic Tight Junctions

**FIGURE 7:**
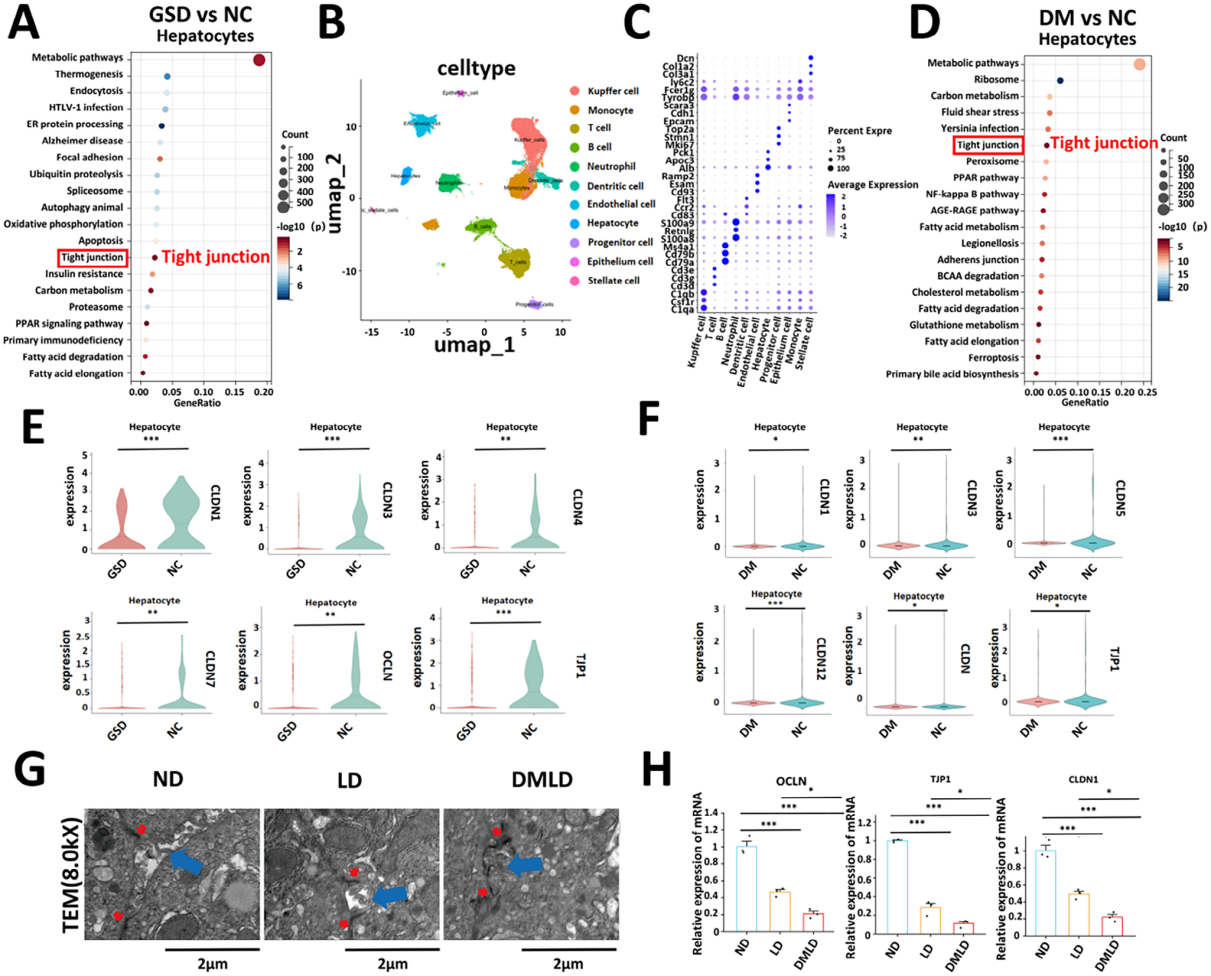
Diabetes promotes the release of NETs into bile by disrupting the tight junctions of liver cells. (A) KEGG enrichment analysis of differentially expressed genes in liver cells from normal control group and patients with gallstones; (B) UMAP visualization of liver tissue cells from normal control group and insulin resistant mice; (C) marker genes used to differentiate various cell types; (D) KEGG enrichment analysis of differentially expressed genes in liver cells from normal control group and insulin resistant mice; (E) comparison of tight junction‐related gene expression in liver cells between normal control group and patients with gallstones; (F) comparison of tight junction‐related gene expression in liver tissues between normal control group and insulin resistant mice; (G) transmission electron microscopy images of tight junctions in liver cells from mice fed a normal diet (ND), lithogenic diet (LD), and diabetes‐induced lithogenic diet (DMLD) (tight junctions, red; bile canaliculi, blue) (*n* = 6 per group); (H) expression analysis of OCLN, TJP1, and CLDN1 genes in liver tissues from each group of mice (*n* = 6 per group). Student's *t*‐test was used for comparisons between two groups, and one‐way ANOVA was used for comparisons among three or more groups. *P < 0.05 was considered statistically significant, **P < 0.01, ***P < 0.001.

Our previous findings demonstrated that diabetes promotes the formation of hepatic NETs through CXCR2 activation in neutrophils. We also observed that NETs formation in the bile of diabetic patients and diabetic mice was markedly increased. This raised the question: how do NETs enter the bile? To explore this mechanism, Kyoto Encyclopedia of Genes and Genomes (KEGG) enrichment analysis was performed using single‐cell sequencing data to identify DEGs in hepatocytes from the GSD and NC groups. The DEGs were significantly enriched in the tight junction signaling pathway (Figure 7A). In parallel, UMAP visualization was conducted using the GEO dataset GSE239612, which contains single‐cell RNA sequencing data from insulin‐resistant and normal mice (Figure 7B,C). Eleven distinct hepatic cell types were identified. KEGG enrichment analysis of DEGs between liver cells from insulin‐resistant and normal mice revealed that these genes were also significantly enriched in the tight junction signaling pathway (Figure 7D). We further analyzed the expression of tight junction‐related genes in hepatocytes from the GSD and NC groups. Compared with the NC group, the expression of tight junction‐related genes in hepatocytes from gallstone patients was significantly reduced (Figure 7E). Interestingly, the expression of tight junction‐related genes in hepatocytes from insulin resistant mice was also markedly decreased compared with that in the control group (Figure 7F). Immunofluorescence evaluation revealed a progressive impairment of tight junction integrity in human liver tissues across groups, with the GSD group showing disruption compared to NC controls, which was exacerbated in the DMSD group (Figure S6A). To directly visualize hepatocellular tight junctions, we examined human liver tissues from the NC, GSD, and DMGSD groups using transmission electron microscopy (TEM). Hepatocyte tight junctions in the DMGSD group were clearly disrupted (Figure S7A). Furthermore, we observed that NETs traversed hepatic sinusoids and entered bile canaliculi in liver tissue by immunogold labeling electron microscopy (Figure S7B). Similarly, examination of liver tissues from mice in each experimental group revealed pronounced disruption of hepatocyte tight junctions in the DMLD group (Figure 7G; Figure S6B). Furthermore, the expression levels of OCLN, TJP1, and CLDN1 in mouse liver tissues were significantly downregulated in diabetic mice (Figure 7H). Collectively, these findings suggest that diabetes may facilitate the entry of NETs into bile by impairing hepatocellular tight junction integrity.

### 3.8 Increased Hepatic NETs Formation Is the Core Pathological Process Through Which Diabetes Disrupts Hepatocellular Tight Junctions

**FIGURE 8:**
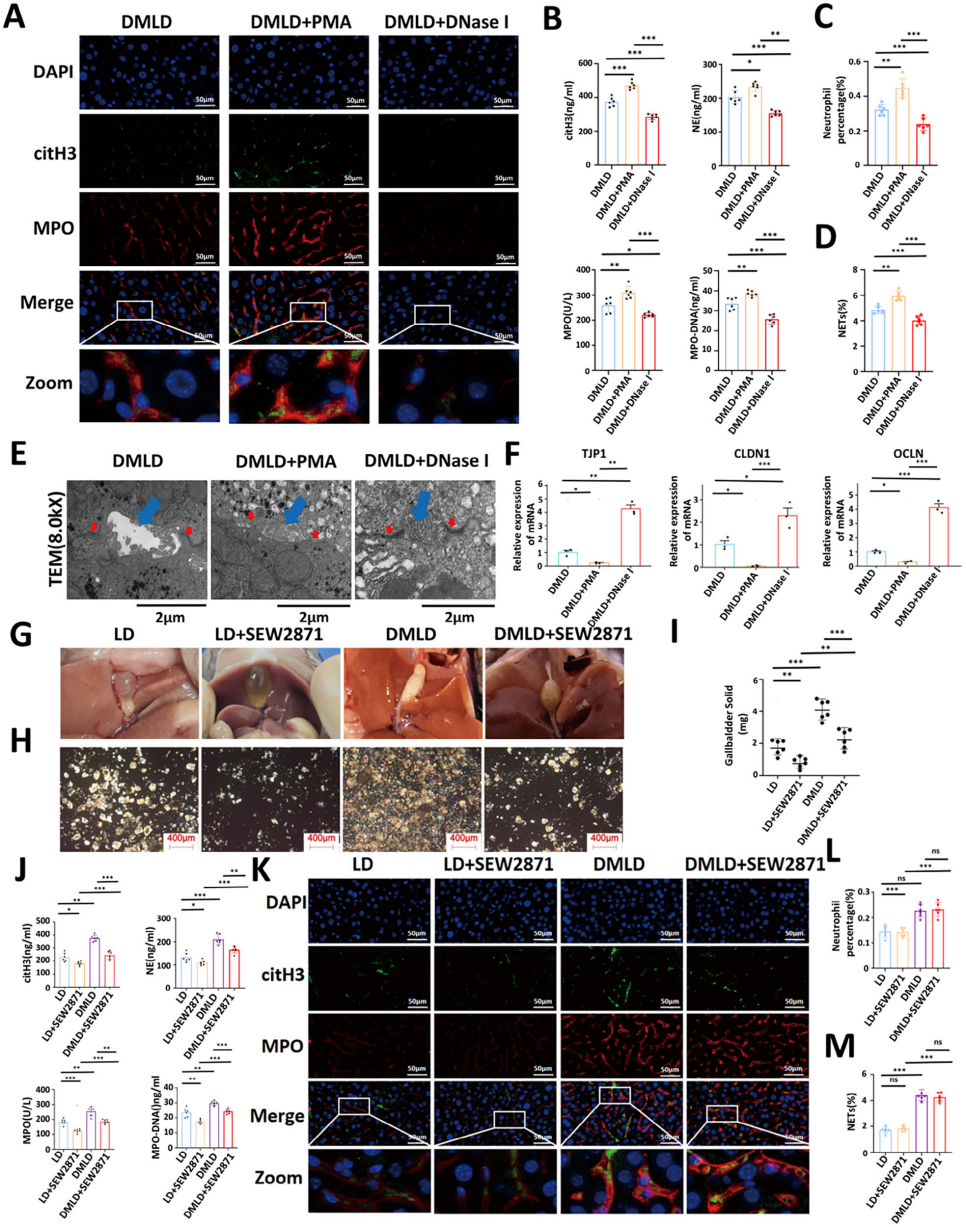
The increase in liver NETs is the core pathological process by which diabetes damages the tight junctions of liver cells. (A) Immunofluorescence images of NETs in liver tissues from mice fed a diabetic lithogenic diet(DMLD), diabetic lithogenic diet with PMA intraperitoneal injection (DMLD+PMA), and diabetic lithogenic diet with DNase I (DMLD+DNase I) intraperitoneal injection (MPO, red; citH3, green; the network structure represents NETs) (*n* = 6 per group); (B) levels of citH3, NE, MPO and MPO‐DNA in bile from each group of mice (*n* = 6 per group); (C) percentage of neutrophils in liver tissues from each group (*n* = 6 per group); (D) percentage of NETs in liver tissues from each group (*n* = 6 per group); (E) transmission electron microscopy images of tight junctions in liver cells from each group (tight junctions, red; bile canaliculi, blue) (*n* = 6 per group); (F) expression analysis of OCLN, TJP1, and CLDN1 genes in liver tissues from each group of mice (*n* = 6 per group); (G) representative images of gallbladders from mice fed a lithogenic diet (LD), lithogenic diet with SEW2871 intraperitoneal injection (LD+SEW2871), diabetic lithogenic diet (DMLD) and diabetic lithogenic diet with SEW2871 intraperitoneal injection (*n* = 6 per group); (H) polarized light microscopy images of bile in gallbladders from each group of mice (*n* = 6 per group); (I) gallstone weights in the gallbladders of each group of mice (*n* = 6 per group); (J) levels of citH3, NE, MPO and MPO‐DNA in bile from each group of mice (*n* = 6 per group); (K) immunofluorescence images of NETs in liver tissues from each group (MPO, red; citH3, green; the network structure represents NETs) (*n* = 6 per group); (L) percentage of neutrophils in liver tissues from each group (*n* = 6 per group); (M) percentage of NETs in liver tissues from each group (*n* = 6 per group). Student's *t*‐test was used for comparisons between two groups, and one‐way ANOVA was used for comparisons among three or more groups. *P < 0.05 was considered statistically significant, **P < 0.01, ***P < 0.001.

To further investigate the specific mechanism by which diabetes disrupts hepatocellular tight junctions during gallstone formation, we intraperitoneally injected PMA into DMLD mice to promote NETs formation, and inhibited NETs formation using DNase I. The experimental results demonstrated that PMA administration significantly increased both the proportion of neutrophils and the formation of NETs in the livers of DMLD mice, whereas DNase I treatment markedly reduced these parameters (Figure 8A,C,D). Interestigly, NETs formation in the bile increased concomitantly with enhanced hepatic NETs generation and decreased following its inhibition (Figure 8B). TEM revealed that hepatocellular tight junctions were markedly disrupted in PMA‐treated mice, while DNase I effectively preserved tight junction integrity (Figure 8E). Moreover, the expression of OCLN, TJP1, and CLDN1 in liver tissues was significantly downregulated in PMA‐treated mice and upregulated following DNase I treatment (Figure 8F). These findings indicate that during diabetes‐induced gallstone formation, disruption of hepatocellular tight junctions occurs in parallel with increased hepatic NETs content. NETs are complex structures containing various proteases and histones. Which key components within NETs are primarily responsible for cleaving and disrupting tight junctions? Previous research has indicated that proteases such as neutrophil elastase (NE), myeloperoxidase (MPO), and matrix metalloproteinase‐9 (MMP9) released from NETs can contribute to cellular and tissue damage [39]. To investigate this, we inhibited NETs‐associated proteases using the NE inhibitor sivelestat, the MPO inhibitor AZD5904, and the MMP9 inhibitor SB‐3CT, respectively, and examined hepatocellular tight junction integrity along with the expression of tight junction‐associated proteins ZO‐1, Occludin, and Claudin‐1. Our results showed that both sivelestat and AZD5904 effectively attenuated NETs‐induced disruption of hepatocellular tight junctions. Moreover, combined inhibition with sivelestat and AZD5904 almost completely rescued the damage to tight junctions caused by NETs (Figure S8A,B). These findings suggest that NE and MPO act synergistically within NETs to degrade and disrupt hepatocellular tight junctions, representing a key mechanism underlying NETs‐mediated liver barrier injury.

SEW2871 has been reported to effectively protect hepatocellular tight junctions and prevent their structural damage [40]. To verify its protective effect in our model, we intraperitoneally injected SEW2871 into mice fed an LD and a DMLD. SEW2871 treatment markedly reduced gallstone formation induced by both LD and DMLD (Figure 8G–I). We next measured NETs‐related indicators in mouse bile and found that SEW2871 significantly suppressed NETs formation in bile (Figure 8J). However, analysis of mouse liver tissues revealed that SEW2871 had no observable effect on either the proportion of hepatic neutrophils or the extent of NETs formation within the liver (Figure 8K–M). These results indicate that, during diabetes‐induced gallstone formation, diabetes facilitates the destruction of hepatocellular tight junctions through the activation of hepatic NETs, thereby enabling the translocation of NETs into bile.

The classical mechanism of gallstone formation involves excessive hepatic cholesterol secretion, leading to biliary cholesterol supersaturation [41]. To determine whether diabetes‐induced NETs formation and disruption of hepatocellular tight junctions alter the cholesterol saturation index (CSI) in bile, we measured the concentrations of bile acids, cholesterol, and phospholipids in human bile samples from the NC, GSD, and DMGSD groups. Compared with the NC group, both the GSD and DMGSD groups exhibited significantly decreased concentrations of bile acids and phospholipids, significantly increased cholesterol levels, and a higher biliary CSI. However, no significant differences in these parameters were observed between the GSD and DMGSD groups (Figure S9A–D). Similarly, in mice, the concentrations of bile acids, cholesterol, and phospholipids were measured in bile from the ND, LD, and DMLD groups. Consistent with the findings in human bile, both the LD and DMLD groups showed markedly reduced bile acid and phospholipid concentrations, elevated cholesterol levels, and an increased biliary CSI compared with the ND group, with no significant differences between the LD and DMLD groups (Figure S9E–H). We also examined the expression of well‐characterized gallstone susceptibility genes in both animal models and human clinical samples. Our results demonstrated no significant differences in the expression profiles of these genes between the group with diabetes complicated by gallstones and the group with gallstones alone (Figure S9I,J). Because diabetes promotes NETs formation and disrupts hepatocellular tight junctions, we next examined whether these effects influence bile flow. Total bile output was measured in mice at different time points, revealing that bile output was significantly greater in the LD group than in the ND group, and further increased in the DMLD group relative to the LD group (Figure S9K). Collectively, these results suggest that diabetes enhances total bile output but does not alter bile composition or the biliary CSI. Therefore, during diabetes‐induced gallstone formation, NETs contribute to gallstone pathogenesis independently rather than synergistically with changes in bile lipid composition.

### 3.9 Sarcosine Inhibits Diabetes‐Induced Gallstone Formation by Suppressing CXCR2 Expression

**FIGURE 9:**
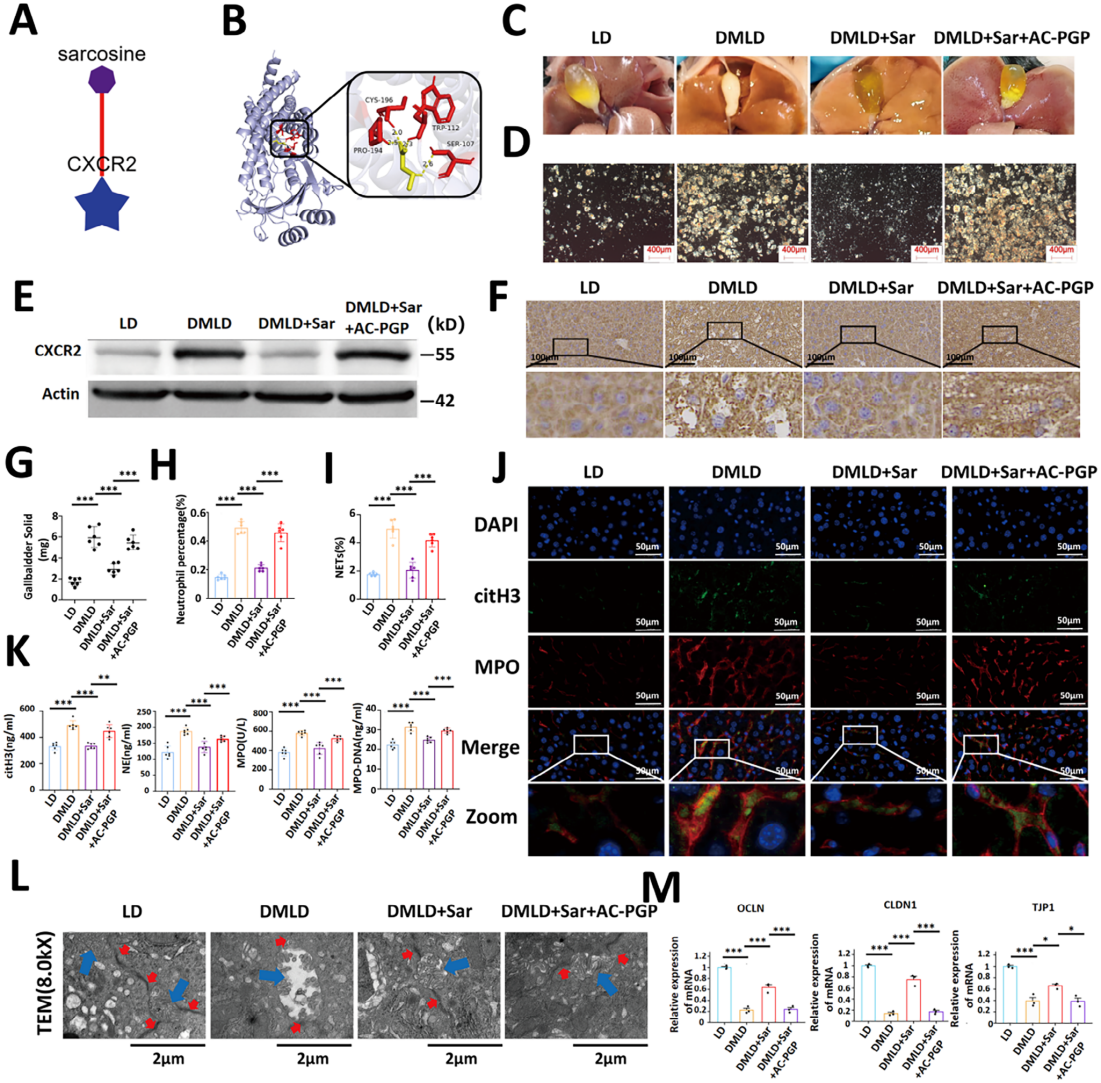
Sarcosine inhibits diabetes‐mediated gallstone formation by suppressing CXCR2 protein expression. (A) Prediction of CXCR2‐targeting drugs using the BATMAN‐TCM database; (B) molecular docking of sarcosinewith CXCR2; (C) representative images of gallbladders from mice fed a lithogenic diet(LD), diabetes‐induced lithogenic diet (DMLD), diabetes‐induced lithogenic diet with intraperitoneal injection of sarcosine (DMLD+Sar), and diabetes‐induced lithogenic diet with intraperitoneal injection of sarcosine plus Ac‐PGP (*n* = 6 per group); (D) polarized light microscopy images of bile in the gallbladders from each group of mice (*n* = 6 per group); (E) western blot analysis of CXCR2 expression in mouse liver tissues (*n* = 6 per group); (F) immunohistochemical staining of CXCR2 in mouse liver tissues (*n* = 6 per group); (G) gallstone weights in the gallbladders of each group (*n* = 6 per group); (H) percentage of neutrophils in liver tissues from each group (*n* = 6 per group); (I) percentage of NETs in liver tissues from each group (*n* = 6 per group); (J) immunofluorescence images of NETs in liver tissues from each group of mice (MPO, red; citH3, green; the network structure represents NETs) (*n* = 6 per group); (K) levels of citH3, NE, MPO and MPO‐DNA in bile from each group of mice (*n* = 6 per group); (L) transmission electron microscopy images of tight junctions in liver cells from each group of mice (tight junctions, red; bile canaliculi, blue) (*n* = 6 per group); (M) expression analysis of OCLN, TJP1, and CLDN1 genes in liver tissues from each group of mice (*n* = 6 per group); Student's *t‐*test was used for comparisons between two groups, and one‐way ANOVA was used for comparisons among three or more groups. *P < 0.05 was considered statistically significant, **P < 0.01, ***P < 0.001.

To identify effective drugs that prevent diabetes‐mediated gallstone formation, we predicted potential bioactive compounds and their targets for CXCR2 using the BATMAN‐TCM database (http://bionet.ncpsb.org.cn/). The analysis suggested that sarcosine may serve as an effective compound targeting the CXCR2 protein (Figure 9A). Molecular docking analysis further indicated that sarcosine directly binds to CXCR2 (Figure 9B). To validate this interaction in vivo, we performed a CXCR2 function–rescue experiment via intraperitoneal injection of Ac‐PGP. Compared with DMLD mice, sarcosine treatment markedly reduced gallstone formation, whereas Ac‐PGP reversed the inhibitory effect of sarcosine on gallstone development in DMLD mice (Figure 9C,D,G). WB and immunohistochemical analyses showed that sarcosine significantly suppressed CXCR2 protein expression in the liver tissues of DMLD mice, and that Ac‐PGP restored CXCR2 expression inhibited by sarcosine (Figure 9E,F). Furthermore, immunofluorescence analysis revealed that sarcosine substantially reduced both the percentage of neutrophils and the formation of NETs in the livers of DMLD mice (Figure 9H–J). Consistently, sarcosine also decreased the levels of NETs‐related markers in bile (Figure 9K). Because our previous results demonstrated that hepatic NETs disrupt hepatocellular tight junctions, we further examined the junctional integrity in this model. The findings indicated that sarcosine protected hepatocellular tight junctions from diabetes‐induced damage, whereas Ac‐PGP reversed this protective effect (Figure 9L,M).

To investigate potential off‐target effects of sarcosine, we employed the CXCR2 antagonist SB225002 administered via intraperitoneal injection, with sarcosine alone and sarcosine combined with SB225002 serving as positive controls. Western blot and immunohistochemical analyses revealed that sarcosine significantly suppressed CXCR2 protein expression in mouse liver tissue, an effect comparable to that of sarcosine combined with SB225002. In contrast, SB225002 alone exhibited a weaker inhibitory effect on CXCR2 expression (Figure S10A,B). Measurement of NETs‐related markers in mouse bile showed that sarcosine markedly reduced the concentrations of citH3, NE, MPO, and MPO‐DNA. Its efficacy was not significantly different from that of SB225002 alone or the combination of sarcosine and SB225002 (Figure S10C). Assessment of NETs formation in liver tissue demonstrated that sarcosine alone significantly inhibited NETs generation. However, both SB225002 and the combination treatment exerted a more pronounced inhibitory effect on NETs formation, with no significant difference observed between these two groups (Figure S10D,E). Notably, sarcosine, SB225002, and their combination achieved comparable efficacy in inhibiting gallstone formation (Figure S10F,G). Furthermore, all three treatments provided an equivalent protective effect on hepatocellular tight junctions in the mice (Figure S10H). Collectively, these findings indicate that sarcosine exhibits minimal off‐target activity and inhibits CXCR2 function primarily by downregulating CXCR2 protein expression. Although sarcosine has a slightly weaker inhibitory effect on CXCR2 function compared with SB225002, it achieves a comparable therapeutic effect in diabetes‐induced gallstone formation.

## 4 Discussion

Gallstone disease, a highly prevalent disorder of the digestive system worldwide, has long drawn attention because of its strong association with metabolic abnormalities [42]. As one of the most common metabolic disorders, diabetes mellitus frequently coexists with gallstone disease, suggesting the presence of unelucidated pathophysiological links between these two conditions [43]. By integrating analyses from the NHANES database, MR validation, animal experiments, and molecular mechanism studies, this work systematically revealed—across both population‐based and experimental dimensions—a novel pathway through which diabetes regulates the formation and degradation of NETs in the hepatic liver–bile barrier, thereby promoting gallstone formation. These findings provide new theoretical insights and identify potential molecular targets for the prevention and treatment of metabolism‐related gallstone disease.

At the population level, our analysis of 3972 participants from the 2017–2020 NHANES database showed a significantly higher incidence of gallstone disease among patients with diabetes mellitus. Even after adjusting for multiple confounding factors, including age, sex, and ethnicity, diabetes remained independently and positively associated with gallstone risk. This result aligns with previous research identifying metabolic comorbidities as risk factors for gallstone disease [44]. Furthermore, MR analysis verified a significant positive causal association between both fasting insulin and type 2 diabetes mellitus (T2DM) and gallstone disease across five statistical models. These results are consistent with the findings of Yan et al. [45]. Additionally, our analysis suggested that T2DM may increase susceptibility not only to gallstone formation but also to cholecystitis, and that elevated serum insulin levels in T2DM patients likely play a crucial role in this process. Collectively, these findings provide genetic and epidemiological evidence supporting a causal role of diabetes mellitus in gallstone pathogenesis.

We employed multiple complementary approaches—including differential gene expression analysis from the GEO database, network pharmacology prediction, and MR analysis—and identified CXCR2 as a key intersecting gene shared between diabetes mellitus and gallstone disease. Analysis of clinical samples further revealed that CXCR2 levels in the peripheral blood of patients with both diabetes mellitus and gallstone disease were significantly higher than those in patients with gallstone disease alone and healthy controls, and these levels were positively correlated with blood glucose concentration. MDS further confirmed that glucose could form a stable complex with the CXCR2 protein: specifically, hydrogen bonds were established with CYX‐196 of CXCR2, and van der Waals interactions occurred with residues such as TRP‐104. These findings suggest that a hyperglycemic environment may directly modulate CXCR2 expression or activity. Consistently, animal experiments demonstrated markedly increased hepatic CXCR2 expression in mice with both diabetes and gallstones, accompanied by significantly greater gallstone weight and incidence compared with mice fed only a lithogenic diet. Conversely, knockdown of hepatic CXCR2 using adeno‐associated virus (AAV) abolished the diabetes‐induced promotion of gallstone formation.

scRNA‐seq of human liver tissues was conducted in this study to determine the cellular localization of CXCR2 within the liver. The results revealed that CXCR2 was predominantly expressed in neutrophils, and its expression level in hepatic neutrophils was significantly higher in patients with both diabetes mellitus and gallstone disease. In a diabetes‐related study by Diana et al., macrophages and pancreatic β‐cells were shown to secrete the chemokines CXCL1 and CXCL2, which recruit CXCR2‐expressing neutrophils from the bloodstream to pancreatic islets. Early administration of CXCR2 antagonists to block neutrophil recruitment was therefore found to inhibit the progression of diabetes in later stages [37]. Based on this evidence, we propose that CXCR2 expression in neutrophils represents a key mechanism by which diabetes mellitus promotes gallstone formation.

Enrichment analysis revealed that the DEGs in neutrophils from the GSD group and those with high CXCR2 expression were significantly enriched in the “neutrophil degranulation” pathway—a critical process in the formation of NETs. NETs, extracellular web‐like structures released by activated neutrophils, have recently been shown to participate in the nucleation and aggregation phases of gallstone formation [26, 46]. In chronic obstructive pulmonary disease, CXCR2 has been implicated in NETs formation and has been shown to promote NETs generation through neutrophil chemotaxis and PAD4‐mediated pathways, thereby contributing to colitis pathogenesis [47, 48]. Evidence from clinical samples and animal experiments in the present study demonstrated that diabetes exacerbates the NETs secretion induced by a lithogenic diet, and that this effect is positively correlated with CXCR2 expression. Importantly, treatment with NETs inhibitors markedly reduced gallstone formation, whereas administration of the NET inducer PMA reversed the inhibitory effect of CXCR2 knockdown on gallstone formation, thereby directly confirming the causal relationship by which CXCR2 promotes gallstone development through the induction of NET formation.

Although previous studies have suggested that the formation of hepatic NETs can promote gallstone development, the mechanism by which NETs enter the biliary system from the liver has remained unclear [49]. This study, for the first time, demonstrates that damage to the hepatic liver–bile barrier by NETs constitutes a critical step enabling their entry into bile and subsequent promotion of gallstone formation. The hepatic liver–bile barrier, which separates the liver's blood circulation from the biliary tract, relies on the integrity of tight junctions between hepatocytes for its stability [50]. Single‐cell sequencing combined with KEGG enrichment analysis revealed that the DEGs of hepatocytes in patients with gallstones disease and in insulin‐resistant mice were significantly enriched in the tight junction signaling pathway, with tight junction‐related genes markedly downregulated. Transmission electron microscopy further confirmed that diabetes causes severe disruption of hepatocellular tight junctions and that increased NETs exacerbate this damage. Moreover, in vitro experiments demonstrated that NETs impair tight junction integrity through the synergistic action of NE and MPO. Notably, treatment with the tight junction–protective agent SEW2871 significantly inhibited gallstone formation in DMLD, and this protective effect was independent of NETs generation, acting instead by preserving the hepatic liver–bile barrier to prevent NETs from entering bile. These findings suggest that, in the diabetic state, excessive hepatic NETs do not directly enter the bile but rather open a “pathway” by damaging hepatocellular tight junctions, thereby facilitating their entry into the biliary system and ultimately contributing to gallstone assembly within bile.

Considering that disruption of the hepatic liver–bile barrier may also increase the leakage of cholesterol and bile acids, we measured bile biochemical parameters, including cholesterol concentration, bile acid concentration, and the CSI. As anticipated, NETs‐induced injury to the hepatic liver–bile barrier led to increased cholesterol leakage; however, it was accompanied by elevated excretion of bile acids and phospholipids, resulting in no significant change in the CSI. Meanwhile, although increased bile flow is conventionally considered protective against gallstone formation by reducing cholesterol retention time, our findings in the diabetic context suggest a more complex scenario. The concomitant NETs‐induced liver–bile barrier damage and enhanced crystal nucleation may override the protective effects of accelerated flow. This paradoxical effect highlights the unique pathophysiology of diabetes‐associated gallstone disease, where inflammatory mechanisms may dominate over traditional lithogenic factors. We further analyzed the protein expression of gallstone susceptibility genes in liver tissues between the simple gallstone group and the gallstone group complicated with diabetes in both animal models and human samples, and found no significant differences. This supports our conclusion that in our experimental system, diabetes does not appear to exacerbate gallstone formation through the traditional cholesterol‐based pathway. Therefore, we conclude that the leakage of NETs, rather than cholesterol, constitutes the principal factor contributing to gallstone formation in the diabetic state.

To exclude the possibility that NETs in bile originate from neutrophils within or infiltrating the gallbladder wall, we performed immunohistochemical staining for NETs markers (MPO and CitH3) and inflammatory cell counting on gallbladder tissues from both human subjects and animal models. The results showed no statistically significant difference in gallbladder NETs levels between the simple gallstone group and the gallstone‐with‐diabetes group. This finding further supports our core conclusion that diabetes exacerbates gallstone formation primarily by promoting the release of NETs from the liver, rather than through NETs originating from the gallbladder itself. If the gallbladder were the major source, higher NETs levels would be expected in the gallstone‐with‐diabetes group, which was not the case.

Based on the aforementioned mechanism, we further investigated potential therapeutic agents targeting CXCR2. Screening through the BATMAN‐TCM database identified sarcosine as a potential regulator of CXCR2. Molecular docking analysis confirmed that sarcosine could directly bind to the CXCR2 protein. In vivo experiments demonstrated that sarcosine treatment markedly reduced hepatic CXCR2 expression in DMLD, inhibited NETs formation, preserved hepatocellular tight junctions, and consequently decreased gallstone formation. In contrast, the CXCR2 agonist Ac‐PGP reversed these protective effects, confirming that sarcosine exerts its therapeutic action by suppressing CXCR2 function. The CXCR2 functional inhibitor SB225002 was used as a positive control to evaluate the inhibitory effect of sarcosine on gallstone formation in DMLD mice [51]. We observed that sarcosine suppressed CXCR2 expression but only partially inhibited NETs generation downstream of CXCR2. Conversely, SB225002 primarily inhibited NETs generation downstream of CXCR2 without significantly affecting CXCR2 protein expression. These findings highlight the therapeutic advantage of sarcosine in diabetes‐associated gallstone disease: it moderately inhibits NETs formation to mitigate gallstone development while avoiding excessive suppression of NETs activity, which could predispose to systemic infections. Overall, these results not only identify sarcosine as a promising candidate for the prevention and treatment of diabetes‐related gallstone disease but also provide experimental evidence supporting an intervention strategy targeting the CXCR2–NETs axis.

For the targeted drug sarcosine that we have identified, we believe it has a promising clinical application prospect. Sarcosine, as an endogenous metabolite, has demonstrated significant potential in the treatment of various diseases such as schizophrenia, tumors, and sarcopenia. Moreover, current clinical studies have shown that the long‐term use of lysine is safe, and it has no significant adverse effects on metabolic parameters such as weight, BMI, and blood pressure [52]. In the treatment of patients with schizophrenia, sarcosine is also considered to be a drug with good patient tolerance and a very low incidence of adverse reactions [53]. Although sarcosine is currently mainly used for mental disorders, we believe it has broader development prospects. Oral solid preparations are currently the most practical administration method. The current dosage for treating mental disorders is usually 2 g per day [54], and new nano‐delivery systems provide a broader prospect for future clinical applications.

Despite the valuable insights gained, this study has certain limitations. First, the therapeutic effect of sarcosine requires further validation through large‐scale animal experiments and clinical trials. In addition, our study aims to preliminarily explore the impact of diabetes itself—independent of obesity—on the pathogenesis of gallstone. Therefore, a cohort of non‐obese diabetic patients was selected to control for confounding factors. This approach, however, imposes limitations on patient selection, as it excludes the typical population of obese patients (BMI ≥ 30 kg/m^2^) with both diabetes and gallstones. We also emphasize that whether the mechanisms identified in this study (such as NETs formation) play a dominant role in obese diabetic patients remains to be validated by future research. Nonetheless, these limitations do not diminish the overall significance of our findings. Through multi‐level validation at the population, animal, and molecular levels, this study establishes a novel pathological mechanism whereby diabetes‐induced upregulation of CXCR2 promotes NETs formation and increases the risk of gallstone development. Collectively, these findings offer a new perspective for elucidating the pathophysiological mechanisms underlying metabolic‐related gallstone disease.

## 5 Conclusion

This study conducted a comprehensive, multi‐dimensional analysis integrating population data, animal models, and molecular mechanism investigations to systematically elucidate the pathological association between diabetes mellitus and gallstone formation. Both the NHANES database analysis and the MR study confirmed that diabetes is an independent risk factor for gallstone disease and demonstrated a positive causal relationship between diabetes and gallstone formation. Mechanistically, diabetes upregulates CXCR2 expression, and elevated CXCR2 activity stimulates neutrophil activation and NET formation. Excessive NETs disrupt hepatocellular tight junctions, thereby compromising the hepatic liver–bile barrier and facilitating the entry of NETs into bile, which in turn promotes gallstone formation. Additionally, animal experiments revealed that sarcosine suppresses CXCR2 expression, inhibits NETs formation, preserves the integrity of the hepatic liver–bile barrier, and ultimately mitigates diabetes‐induced gallstone formation. Collectively, these findings provide a theoretical foundation and potential therapeutic direction for the clinical prevention and treatment of diabetes‐associated gallstone disease.

## Author Contributions

Chao Shi, Shuo Feng and Tianming Liu contributed equally to this work. Chao Shi, Shuo Feng, and Tianming Liu: Writing‐ Original draft preparation and complete the animal experiments. Ziang Meng, Yi Zheng, and Zhenghao Huang: Software and Data curation. Tong Wang and Bojian Zhang: Methodology. Dongbo Xue: Conceptualization. Xianzhi Meng and Biao Ma: Funding acquisition, Writing the Reviewing and Editing

## Funding

This work was supported by grants from the National Natural Science Foundation of China (82270598), the Outstanding Youth Foundation of the First Affiliated Hospital of Harbin Medical University (No. 2021J12, BM) and the Open Fund of Key Laboratory of Hepatosplenic Surgery, Ministry of Education, Harbin, China (GPKF202506; GPKF202404).

## Ethics Statement

All human and animal experiments were approved by the Ethics Committee of the First Affiliated Hospital of Harbin Medical University (No. 2025395;No.2024081)

## Conflicts of Interest

The authors declare no conflict of interest.

## Supporting information




**Supporting File**: advs74477‐sup‐0001‐SuppMat.docx.

## Data Availability

The authors state that the data underlying the findings of this study are accessible within the paper. In the event that raw data files in alternative formats are required, they can be obtained from the corresponding author upon a reasonable request.
