# Supplementary material for: Diabetes Mellitus Facilitates Gallstone Formation Through CXCR2‐NETs–Mediated Liver‐Bile Barrier Damage
Source: Adv Sci (Weinh). 2026 Feb 16;13(24):e19500. doi: 10.1002/advs.202519500 (PMC13116217; doi:10.1002/advs.202519500)
Supplement: Supplementary file 1 — Supporting File: advs74477‐sup‐0001‐SuppMat.docx. [file ADVS-13-e19500-s001.docx]

**Supplementary Materials**


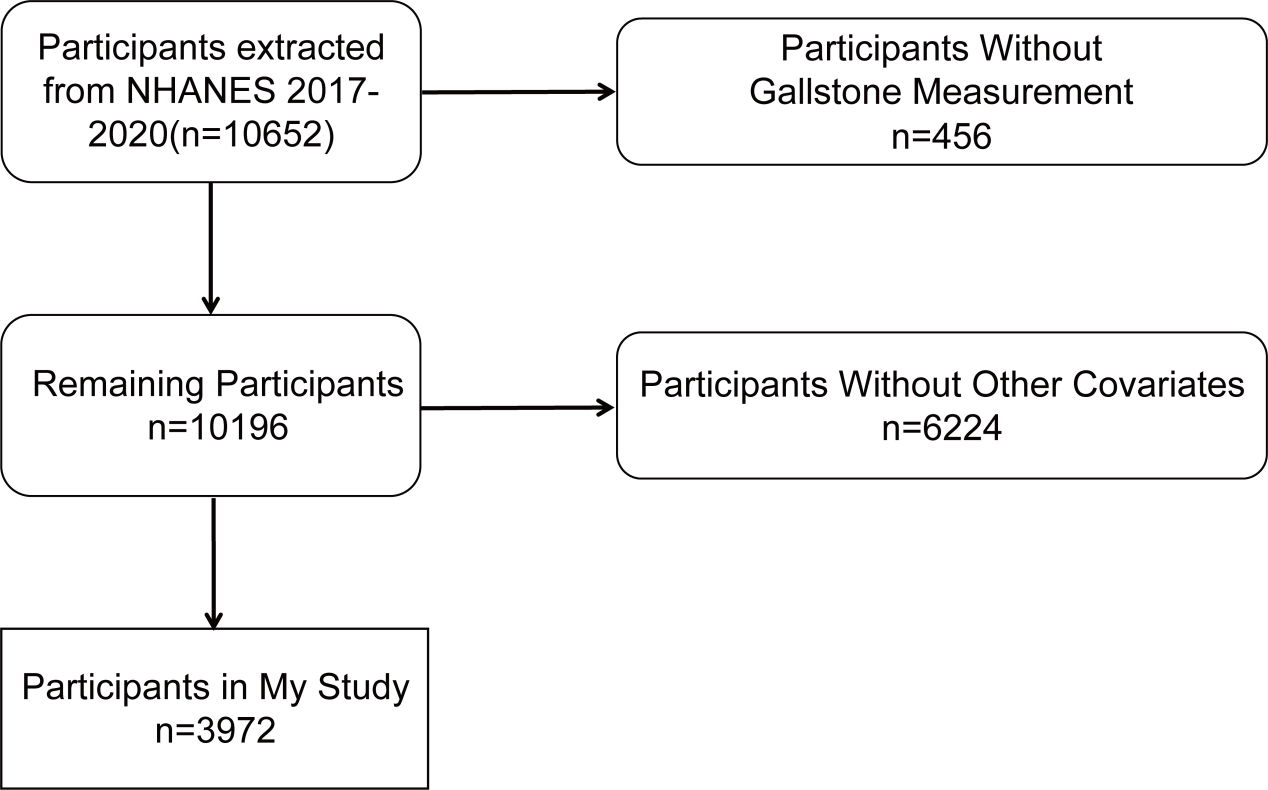


**Supplementary Figure 1.** Flowchart showing the selection of the studied population.





**Supplementary Figure 2.** Mendelian randomization analysis of the causal effect of diabetes on cholelithiasis. A. Forest plot showing the MR effect estimates of individual SNPs on the outcome, with the overall causal estimates from MR Egger and inverse variance weighted methods indicated at the bottom; B. Scatter plot illustrating the relationship between β_IV_ and 1/SE_IV_, featuring a notable outlier and reference lines for MR estimates; C. Scatter plot comparing SNP effects on exposure and outcome, with regression lines fitted by multiple MR methods; D. Leave-one-out sensitivity analysis forest plot displaying the stability of the causal estimate upon the iterative exclusion of each SNP.


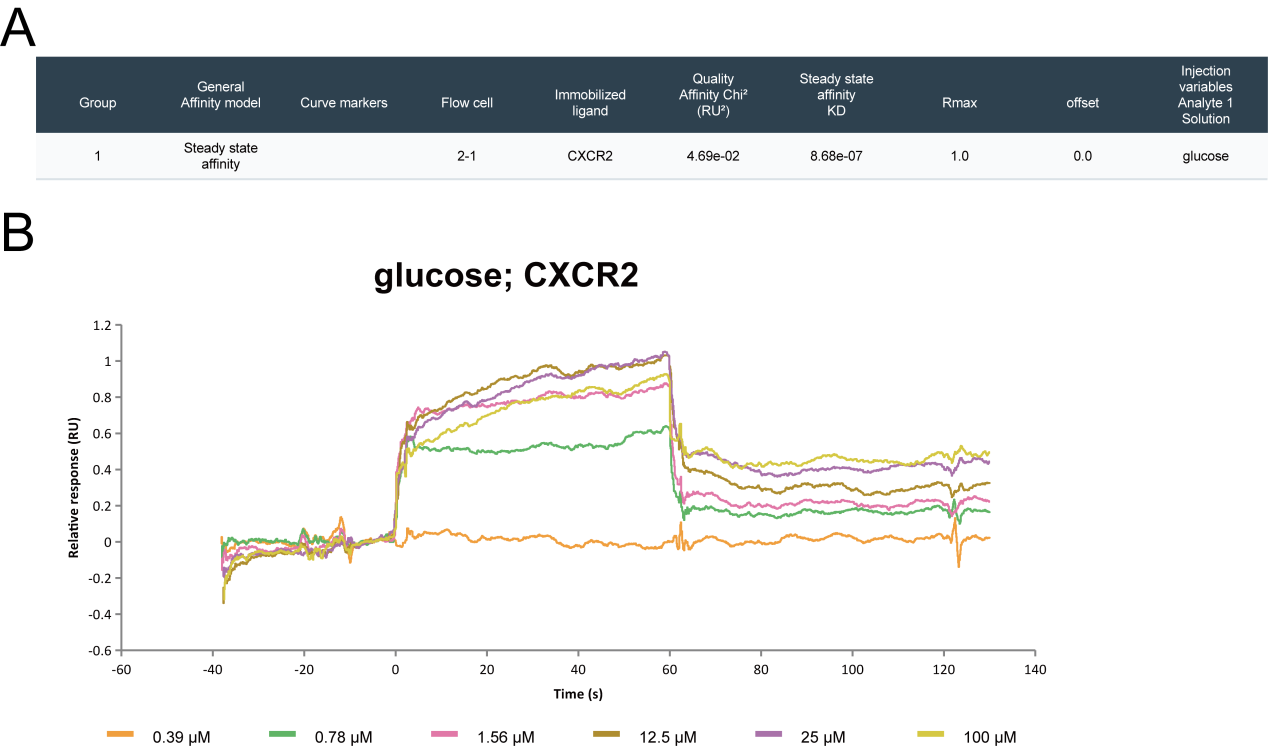


**Supplementary Figure 3.** SPR analysis of glucose to CXCR2. A. Key kinetic and affinity parameters derived from steady-state affinity analysis. The low Chi^2^ value (4.69e-02) indicates excellent fitting of the data to a 1:1 binding model. The equilibrium dissociation constant (KD) was determined to be 0.87 µM; B. Real-time sensorgrams showing the binding of glucose at increasing concentrations (0.39, 0.78, 1.56, 12.5, 25, and 100 µM) to immobilized CXCR2. The arrow indicates the start of the dissociation phase.


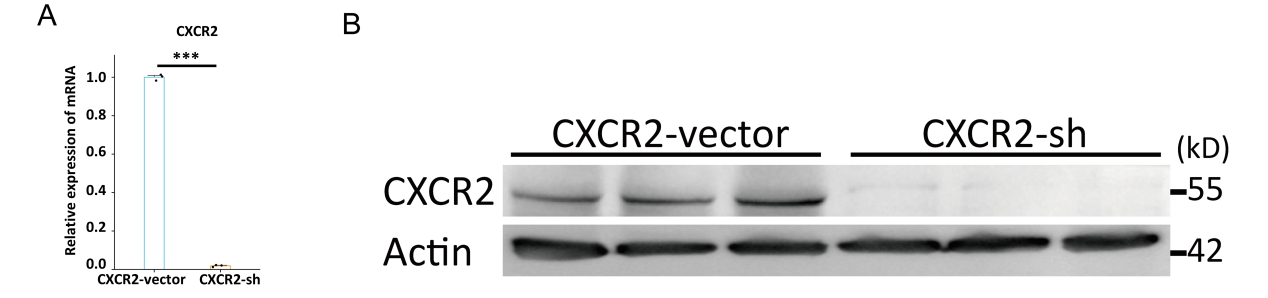


**Supplementary Figure 4.** Assessment of CXCR2 gene knockdown efficiency. A. Assessment of mRNA expression in CXCR2 viral injection(CXCR2-sh) versus control groups(CXCR2-vector)(n = 3 per group); B. Western blot analysis of CXCR2 protein expression across experimental groups(n = 3 per group). Student’s t-test was used for comparisons between two groups. *P < 0.05 was considered statistically significant, **P < 0.01, ***P < 0.001.


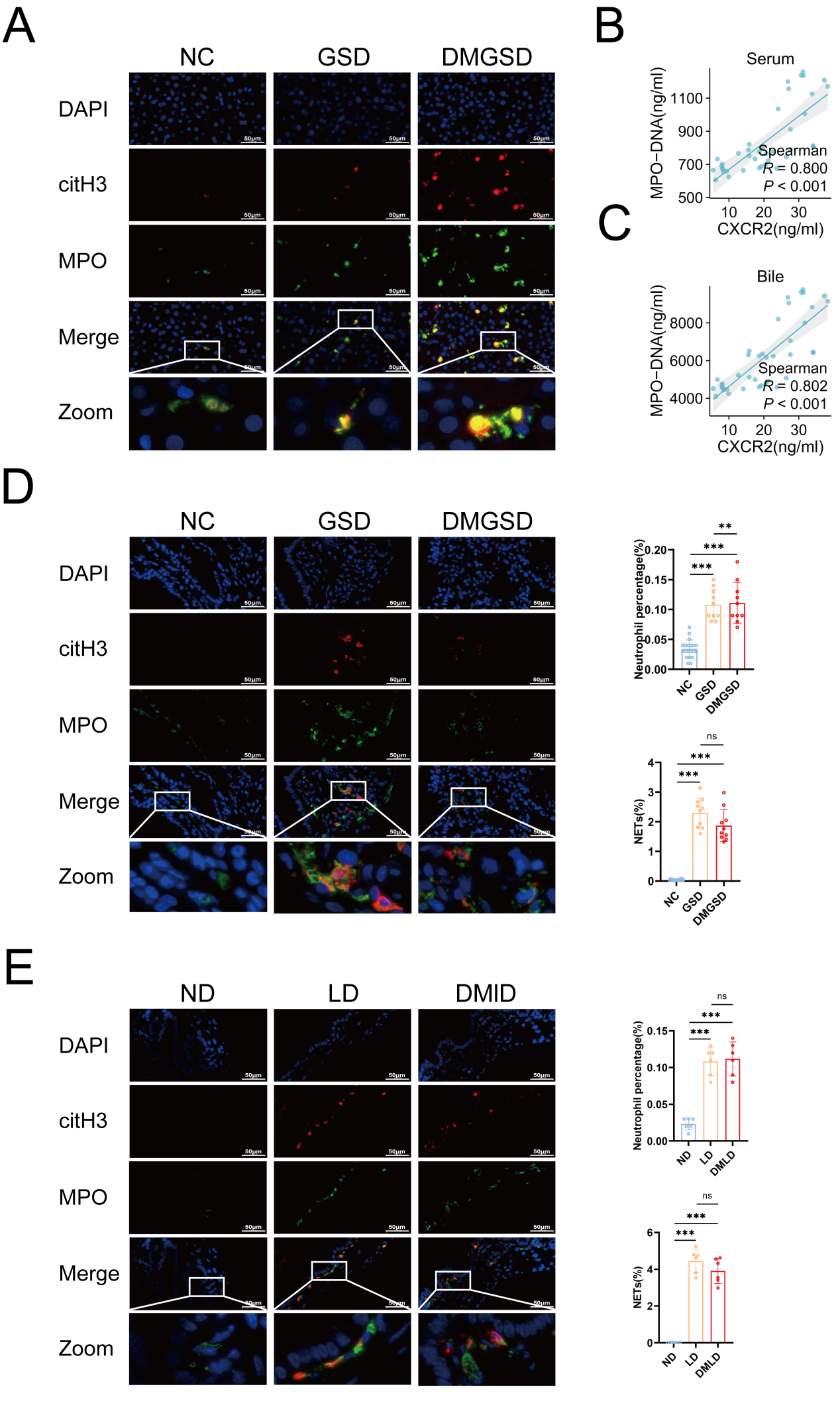


**Supplementary Figure 5.** Measurement of NETs-associated markers. A. Immunofluorescence images of NETs (citH3, red; MPO, green; the network structure represents NETs) in human liver tissues of each group(n = 20 NC group,n = 10 GSD and DMGSD group); B. Correlation analysis between serum CXCR2 levels and serum levels of MPO-DNA(n = 40); C. Correlation analysis between serum CXCR2 levels and levels of MPO-DNA in bile(n = 40); D. Percentage of neutrophils and immunofluorescence images of NETs (citH3, red; MPO, green; the network structure represents NETs) in human gallbladder tissue of each group(n = 20 NC group,n = 10 GSD and DMGSD group); E. Percentage of neutrophils and immunofluorescence images of NETs (citH3, red; MPO, green; the network structure represents NETs) in mouse gallbladder tissue of each group(n = 6 per group).


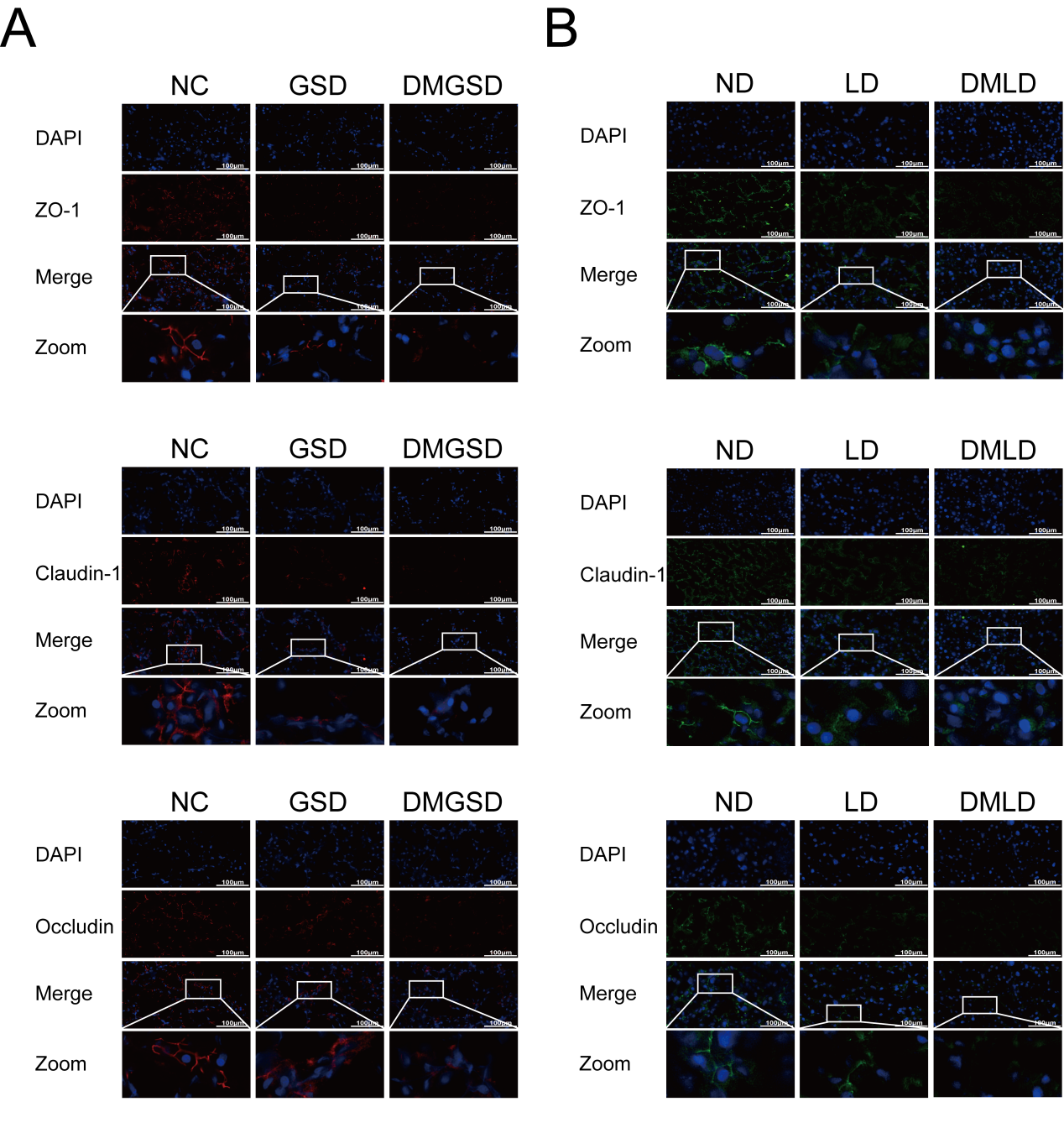


**Supplementary Figure 6.** Immunofluorescence detection of tight junction-associated proteins in the liver. A. Immunofluorescence images of ZO-1,Claudin-1 and Occludin in human liver tissue of each group(n = 20 NC group,n = 10 GSD and DMGSD group); B. Immunofluorescence images of ZO-1,Claudin-1 and Occludin in mouse liver tissue of each group(n = 6 per group).


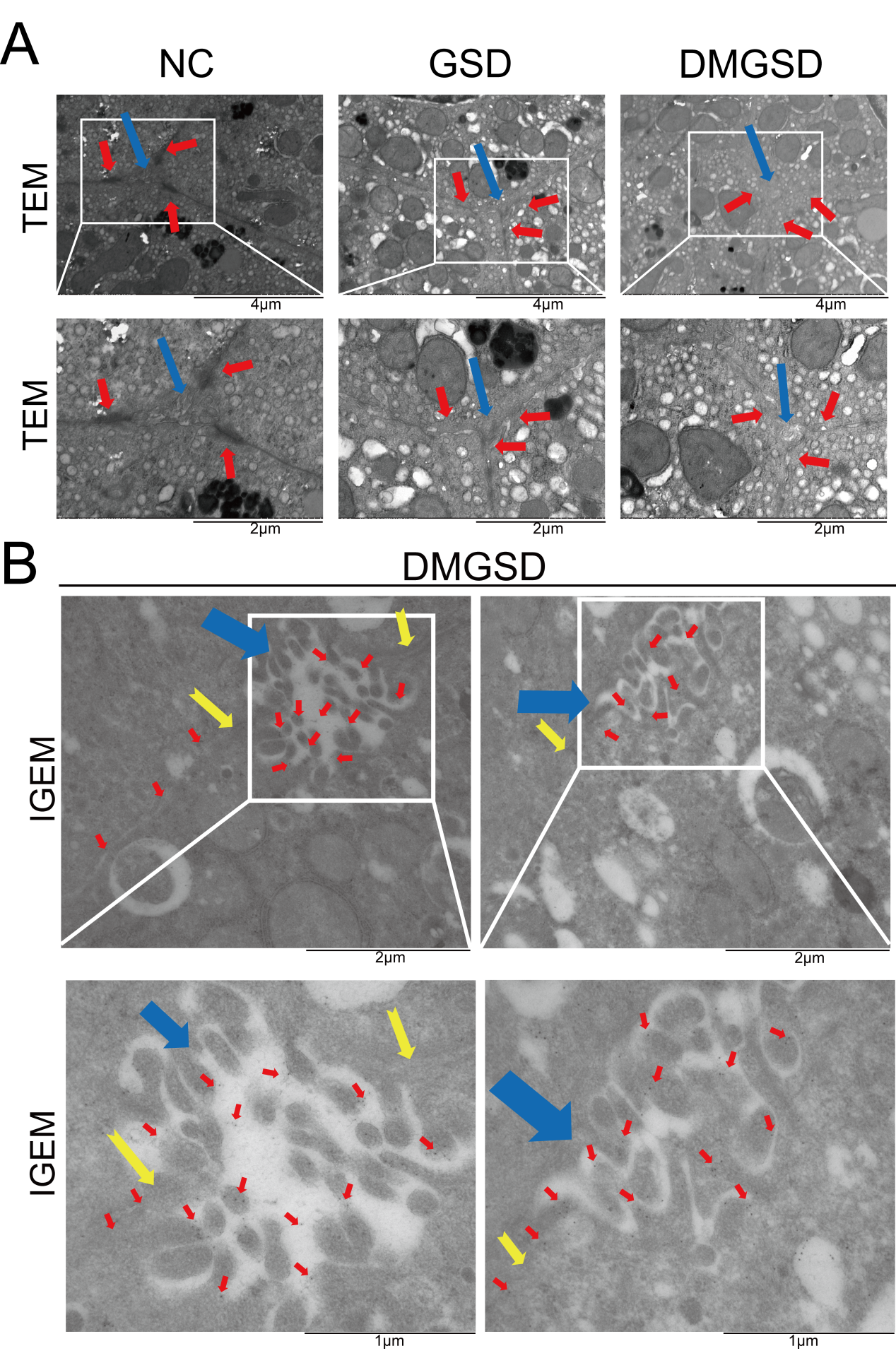


**Supplementary Figure 7.** Transmission electron microscopy images of tight junctions in human liver tissue. A. Transmission electron microscopy images of tight junctions in human liver tissue of normal control group(NC), patients with cholelithiasis(GSD), and patients with diabetes-related cholelithiasis(DMGSD)(tight junctions, red; bile canaliculi, blue)(n = 20 NC group,n = 10 GSD and DMGSD group); B. Iimmunogold labeling electron microscopy images of NETs traversing from sinusoids to bile canaliculi(NETs, red; bile canaliculi, blue; interhepatocytic space, yellow).





**Supplementary Figure 8.** NE and MPO act synergistically within NETs to degrade and disrupt hepatocellular tight junctions. A. Western blot analysis of ZO-1, occludin and claudin-1 protein expression across experimental groups(n = 3 per group); B. Measurement of transepithelial electrical resistance (TEER) in THLE-2 cells across experimental groups. Student’s t-test was used for comparisons between two groups. *P < 0.05 was considered statistically significant, **P < 0.01, ***P < 0.001.


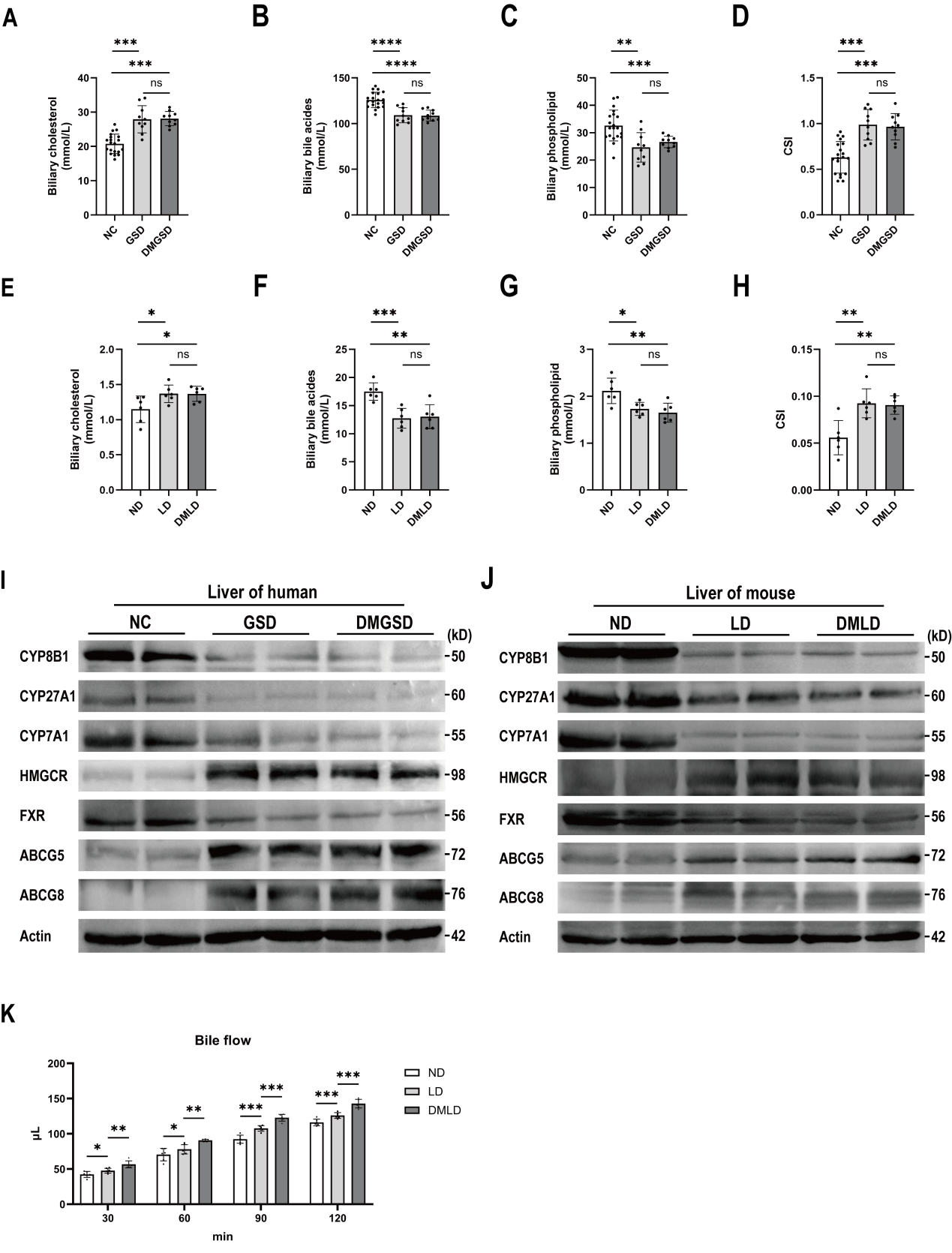


**Supplementary Figure 9.** Parameters of cholesterol metabolism in bile. A. Biliary cholesterol concentration of normal control group(NC), patients with cholelithiasis(GSD), and patients with diabetes-related cholelithiasis(DMGSD)(n = 20 NC group,n = 10 GSD and DMGSD group); B. Biliary bile acides concentration from each group(n = 20 NC group,n = 10 GSD and DMGSD group); C. Biliary phospholipid concentration from each group(n = 20 NC group,n = 10 GSD and DMGSD group); D. Cholesterol Saturation Index rom each group(n = 20 NC group,n = 10 GSD and DMGSD group); E. Biliary cholesterol concentration of mice fed a normal diet(ND), lithogenic diet(LD), or diabetes with lithogenic diet(DMLD)(n = 6 per group); F. Biliary bile acides concentration from each group(n = 6 per group); G. Biliary phospholipid concentration from each group(n = 6 per group); H. Cholesterol Saturation Index rom each group(n = 6 per group); I. Immunoblotting analysis of proteins(CYP8B1, CYP27A1, CYP7A1, HMGCR, FXR, ABCG5 and ABCG8) related to cholesterol and bile acid metabolism in human liver tissues from each group(n = 20 NC group,n = 10 GSD and DMGSD group); J. Immunoblotting analysis of proteins(CYP8B1, CYP27A1, CYP7A1, HMGCR, FXR, ABCG5 and ABCG8) related to cholesterol and bile acid metabolism in mouse liver tissues from each group(n =6 per group); K. Bile flow from each group at different time points(n = 6 per group). Student's t-test was used for comparisons between two groups. *P < 0.05 was considered statistically significant, **P < 0.01, ***P < 0.001.


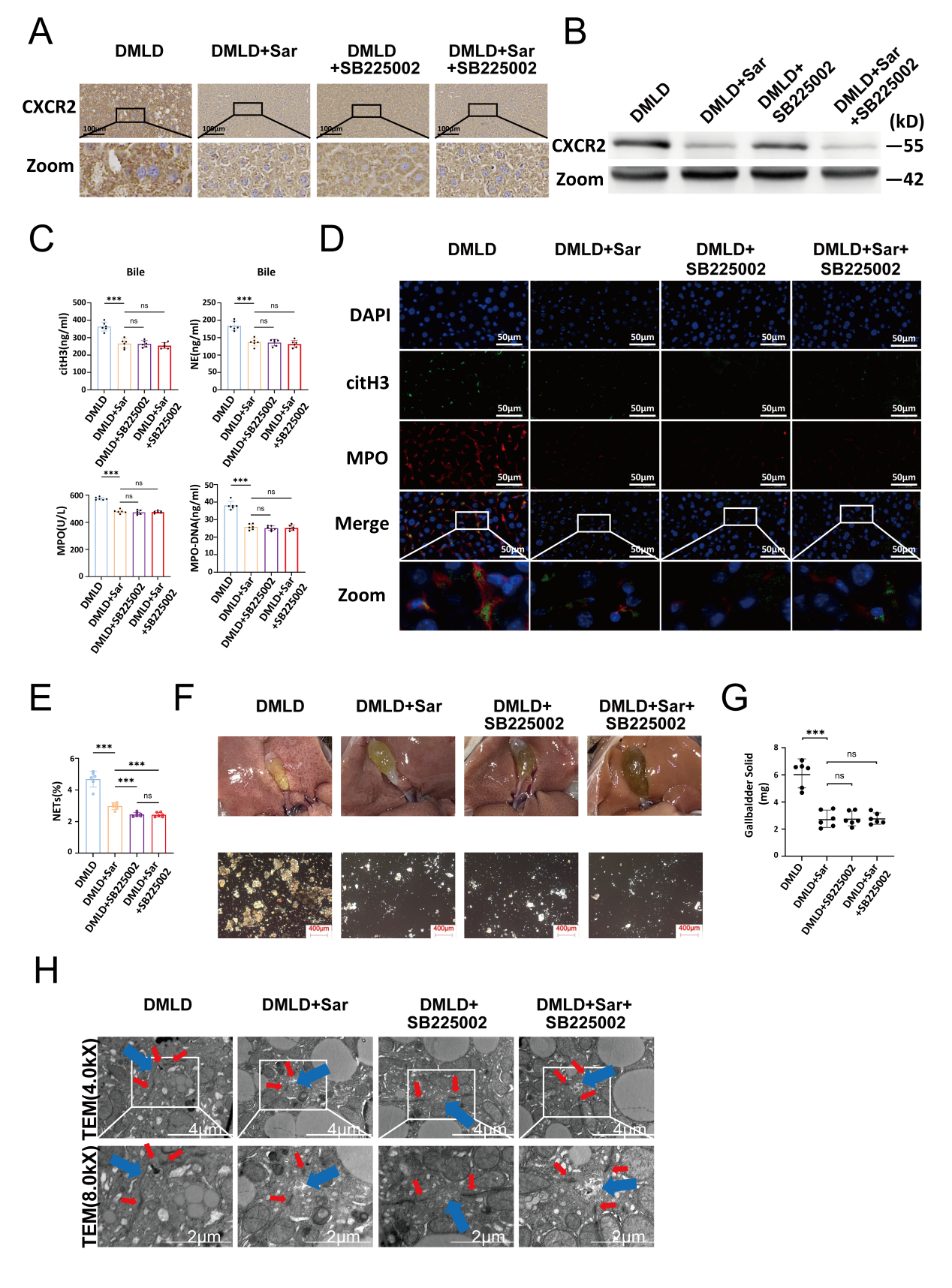


**Supplementary Figure 10.** Assessment of sarcosine off-target effects. A. Immunohistochemical staining of CXCR2 in mouse liver tissues from mice fed a diabetes-induced lithogenic diet(DMLD), diabetes-induced lithogenic diet with intraperitoneal injection of sarcosine(DMLD + Sar), diabetes-induced lithogenic diet with intraperitoneal injection of sb225002(DMLD + SB225002) and diabetes-induced lithogenic diet with intraperitoneal injection of sarcosine and SB225002(DMLD + Sar + SB225002)(n = 6 per group); B. Western blot analysis of CXCR2 expression in mouse liver tissues(n = 6 per group); C. Levels of citH3, NE, MPO and MPO-DNA in bile from each group of mice(n = 6 per group); D. Immunofluorescence images of NETs in liver tissues from each group of mice (MPO, red; citH3, green; the network structure represents NETs)(n = 6 per group); E. Percentage of NETs in liver tissues from each group(n = 6 per group); F. Representative images of gallbladders and Polarized light microscopy images of bile in the gallbladders from each group of mice(n = 6 per group); G. Gallstone weights in the gallbladders of each group(n = 6 per group) ; H. Transmission electron microscopy images of tight junctions in liver cells from each group of mice(tight junctions, red; bile canaliculi, blue)(n = 6 per group). Student’s t-test was used for comparisons between two groups, and one-way ANOVA was used for comparisons among three or more groups. *P < 0.05 was considered statistically significant, **P < 0.01, ***P < 0.001.

**Supplementary Table 1 Description of 3,972 participants included in the present study**

| **variable** | **total** | **Non-gallstone** | **Gallstone** | ***P-*value** |
| --- | --- | --- | --- | --- |
| **FPG** | 6.04(0.05) | 6.00(0.05) | 6.39(0.10) | **0.002** |
| **Age** | 46.12(0.59) | 45.27(0.58) | 54.16(0.98) | **< 0.001** |
| **Poverty** | 3.26(0.05) | 3.27(0.05) | 3.21(0.12) | 0.65 |
| **FSI** | 13.25(0.45) | 12.49(0.41) | 20.35(1.98) | **< 0.001** |
| **HbA1c** | 5.61(0.02) | 5.59(0.02) | 5.86(0.05) | **< 0.001** |
| **METS-IR** | 43.48(0.37) | 42.95(0.38) | 48.49(1.05) | **< 0.001** |
| **HOMA-IR** | 3.88(0.17) | 3.54(0.14) | 7.06(1.11) | **0.003** |
| **SHR** | 0.95(0.00) | 0.95(0.00) | 0.95(0.01) | 0.48 |
| **BMI** | 29.47(0.19) | 29.14(0.18) | 32.65(0.62) | **< 0.001** |
| **Race** |  |  |  | **0.05** |
| Black | 10.08(0.01) | 10.70(1.21) | 4.31(0.89) |  |
| Mexican | 9.01(0.01) | 8.98(1.21) | 9.27(1.26) |  |
| Other | 15.23(0.01) | 15.06(1.22) | 16.79(3.23) |  |
| White | 65.68(0.03) | 65.26(1.97) | 69.64(3.71) |  |
| **Edu** |  |  |  | 0.89 |
| >High school | 91.23(0.03) | 91.25(0.63) | 90.99(1.58) |  |
| ≤High school | 8.77(0.01) | 8.75(0.63) | 9.01(1.58) |  |
| **DM** |  |  |  | **< 0.001** |
| No | 86.33(0.03) | 87.48(0.80) | 75.53(2.29) |  |
| Yes | 13.67(0.01) | 12.52(0.80) | 24.47(2.29) |  |
| **Sex** |  |  |  | **< 0.001** |
| Female | 49.73(0.02) | 46.74(1.10) | 77.85(3.53) |  |
| Male | 50.27(0.02) | 53.26(1.10) | 22.15(3.53) |  |
| **Smoke** |  |  |  | 0.51 |
| No | 83.97(0.03) | 83.82(1.10) | 85.43(2.35) |  |
| Yes | 16.03(0.01) | 16.18(1.10) | 14.57(2.35) |  |
| **Alcohol** |  |  |  | 0.95 |
| Drinker | 92.90(0.03) | 92.89(0.68) | 92.97(1.50) |  |
| Non-drinker | 7.10(0.01) | 7.11(0.68) | 7.03(1.50) |  |

FPG: Fasting Plasma Glucose; FSI: Fasting Serum Insulin; HbA1c: Hemoglobin A1c; METS-IR: Metabolic Score for Insulin Resistance; HOMA-IR: Homeostasis Model Assessment of Insulin Resistance; SHR: Stress Hyperglycemia Ratio; BMI: BodyMass Index; DM: Diabetes Mellitus. Ref: reference. All the results are weighted.

**Supplementary Table 2 Univariate logistic regression analysis of gallstone risk factor in NHANES**

| **character** | **Estimate** | **Std.Error** | **T-value** | ***P-*value** | **OR(95%CI)** |
| --- | --- | --- | --- | --- | --- |
| **FPG** | 0.0973 | 0.027 | 3.5998 | **<0.001** | 1.1022(1.0435-1.1641) |
| **Age** | 0.0329 | 0.0034 | 9.6306 | **<0.0001** | 1.0335(1.0264-1.0407) |
| **Poverty** | -0.0211 | 0.0044 | -0.4543 | 0.6522 | 0.9791(0.8913-1.0756) |
| **FSI** | 0.0102 | 0.0005 | 2.3021 | **0.0268** | 1.0103(1.0012-1.0194) |
| **HbA1c** | 0.2497 | 0.042 | 5.9411 | **<0.0001** | 1.2836(1.1790-1.3975) |
| **METS-IR** | 0.027 | 0.0044 | 6.1682 | **<0.0001** | 1.0274(1.0183-1.0365) |
| **HOMA-IR** | 0.0291 | 0.0086 | 3.3728 | **0.0017** | 1.0295(1.0117-1.0477) |
| **SHR** | -0.462 | 0.6454 | -0.7159 | 0.4783 | 0.6300(0.1708-2.3243) |
| **BMI** | 0.0545 | 0.0079 | 6.8803 | **<0.0001** | 1.0560(1.0392-1.0731) |
| **Race** |  |  |  |  |  |
| Black | ref | ref | ref | ref | ref |
| Mexican | 0.9406 | 0.205 | 4.5879 | **<0.0001** | 2.5614(1.6908-3.8804) |
| Other | 1.0185 | 0.2467 | 4.1281 | **<0.001** | 2.7689(1.6796-4.5647) |
| White | 0.9749 | 0.2258 | 4.318 | **<0.001** | 2.6508(1.6777-4.1884) |
| **Edu** |  |  |  |  |  |
| >High school | ref | ref | ref | ref | ref |
| ≤High school | 0.0327 | 0.2284 | 0.1434 | 0.8867 | 1.0333(0.6510-1.6402) |
| **DM** |  |  |  |  |  |
| No | ref | ref | ref | ref | ref |
| Yes | 0.8167 | 0.1465 | 5.576 | **<0.0001** | 2.2630(1.6827-3.0433) |
| **Sex** |  |  |  |  |  |
| Female | ref | ref | ref | ref | ref |
| Male | -1.3877 | 0.2145 | -6.4691 | **<0.0001** | 0.2496(0.1618-0.3853) |
| **Smoke** |  |  |  |  |  |
| No | ref | ref | ref | ref | ref |
| Yes | -0.1242 | 0.187 | -0.6643 | 0.5104 | 0.8832(0.6050-1.2892) |
| **Alcohol** |  |  |  |  |  |
| Drinker | ref | ref | ref | ref | ref |
| Non-drinker | -0.0128 | 0.2209 | -0.0578 | 0.9542 | 0.9873(0.6315-1.5436) |

FPG: Fasting Plasma Glucose; FSI: Fasting Serum Insulin; HbA1c: Hemoglobin A1c; METS-IR: Metabolic Score for Insulin Resistance; HOMA-IR: Homeostasis Model Assessment of Insulin Resistance; SHR: Stress Hyperglycemia Ratio; BMI: BodyMass Index; DM: Diabetes Mellitus. Ref: reference. All the results are weighted.
